# Genomic footprints of activated telomere maintenance mechanisms in cancer

**DOI:** 10.1038/s41467-019-13824-9

**Published:** 2020-02-05

**Authors:** Lina Sieverling, Chen Hong, Sandra D. Koser, Philip Ginsbach, Kortine Kleinheinz, Barbara Hutter, Delia M. Braun, Isidro Cortés-Ciriano, Ruibin Xi, Rolf Kabbe, Peter J. Park, Roland Eils, Matthias Schlesner, PCAWG-Structural Variation Working Group, Kadir C. Akdemir, Eva G. Alvarez, Adrian Baez-Ortega, Rameen Beroukhim, Paul C. Boutros, David D. L. Bowtell, Benedikt Brors, Kathleen H. Burns, Peter J. Campbell, Kin Chan, Ken Chen, Isidro Cortés-Ciriano, Ana Dueso-Barroso, Andrew J. Dunford, Paul A. Edwards, Xavier Estivill, Dariush Etemadmoghadam, Lars Feuerbach, J. Lynn Fink, Milana Frenkel-Morgenstern, Dale W. Garsed, Mark Gerstein, Dmitry A. Gordenin, David Haan, James E. Haber, Julian M. Hess, Barbara Hutter, Marcin Imielinski, David T. W. Jones, Young Seok Ju, Marat D. Kazanov, Leszek J. Klimczak, Youngil Koh, Jan O. Korbel, Kiran Kumar, Eunjung Alice Lee, Jake June-Koo Lee, Yilong Li, Andy G. Lynch, Geoff Macintyre, Florian Markowetz, Iñigo Martincorena, Alexander Martinez-Fundichely, Matthew Meyerson, Satoru Miyano, Hidewaki Nakagawa, Fabio C. P. Navarro, Stephan Ossowski, Peter J. Park, John V. Pearson, Montserrat Puiggròs, Karsten Rippe, Nicola D. Roberts, Steven A. Roberts, Bernardo Rodriguez-Martin, Steven E. Schumacher, Ralph Scully, Mark Shackleton, Nikos Sidiropoulos, Lina Sieverling, Chip Stewart, David Torrents, Jose M. C. Tubio, Izar Villasante, Nicola Waddell, Jeremiah A. Wala, Joachim Weischenfeldt, Lixing Yang, Xiaotong Yao, Sung-Soo Yoon, Jorge Zamora, Cheng-Zhong Zhang, Benedikt Brors, Karsten Rippe, David T. W. Jones, Lars Feuerbach, PCAWG Consortium, Lauri A. Aaltonen, Federico Abascal, Adam Abeshouse, Hiroyuki Aburatani, David J. Adams, Nishant Agrawal, Keun Soo Ahn, Sung-Min Ahn, Hiroshi Aikata, Rehan Akbani, Kadir C. Akdemir, Hikmat Al-Ahmadie, Sultan T. Al-Sedairy, Fatima Al-Shahrour, Malik Alawi, Monique Albert, Kenneth Aldape, Ludmil B. Alexandrov, Adrian Ally, Kathryn Alsop, Eva G. Alvarez, Fernanda Amary, Samirkumar B. Amin, Brice Aminou, Ole Ammerpohl, Matthew J. Anderson, Yeng Ang, Davide Antonello, Pavana Anur, Samuel Aparicio, Elizabeth L. Appelbaum, Yasuhito Arai, Axel Aretz, Koji Arihiro, Shun-ichi Ariizumi, Joshua Armenia, Laurent Arnould, Sylvia Asa, Yassen Assenov, Gurnit Atwal, Sietse Aukema, J. Todd Auman, Miriam R. R. Aure, Philip Awadalla, Marta Aymerich, Gary D. Bader, Adrian Baez-Ortega, Matthew H. Bailey, Peter J. Bailey, Miruna Balasundaram, Saianand Balu, Pratiti Bandopadhayay, Rosamonde E. Banks, Stefano Barbi, Andrew P. Barbour, Jonathan Barenboim, Jill Barnholtz-Sloan, Hugh Barr, Elisabet Barrera, John Bartlett, Javier Bartolome, Claudio Bassi, Oliver F. Bathe, Daniel Baumhoer, Prashant Bavi, Stephen B. Baylin, Wojciech Bazant, Duncan Beardsmore, Timothy A. Beck, Sam Behjati, Andreas Behren, Beifang Niu, Cindy Bell, Sergi Beltran, Christopher Benz, Andrew Berchuck, Anke K. Bergmann, Erik N. Bergstrom, Benjamin P. Berman, Daniel M. Berney, Stephan H. Bernhart, Rameen Beroukhim, Mario Berrios, Samantha Bersani, Johanna Bertl, Miguel Betancourt, Vinayak Bhandari, Shriram G. Bhosle, Andrew V. Biankin, Matthias Bieg, Darell Bigner, Hans Binder, Ewan Birney, Michael Birrer, Nidhan K. Biswas, Bodil Bjerkehagen, Tom Bodenheimer, Lori Boice, Giada Bonizzato, Johann S. De Bono, Arnoud Boot, Moiz S. Bootwalla, Ake Borg, Arndt Borkhardt, Keith A. Boroevich, Ivan Borozan, Christoph Borst, Marcus Bosenberg, Mattia Bosio, Jacqueline Boultwood, Guillaume Bourque, Paul C. Boutros, G. Steven Bova, David T. Bowen, Reanne Bowlby, David D. L. Bowtell, Sandrine Boyault, Rich Boyce, Jeffrey Boyd, Alvis Brazma, Paul Brennan, Daniel S. Brewer, Arie B. Brinkman, Robert G. Bristow, Russell R. Broaddus, Jane E. Brock, Malcolm Brock, Annegien Broeks, Angela N. Brooks, Denise Brooks, Benedikt Brors, Søren Brunak, Timothy J. C. Bruxner, Alicia L. Bruzos, Alex Buchanan, Ivo Buchhalter, Christiane Buchholz, Susan Bullman, Hazel Burke, Birgit Burkhardt, Kathleen H. Burns, John Busanovich, Carlos D. Bustamante, Adam P. Butler, Atul J. Butte, Niall J. Byrne, Anne-Lise Børresen-Dale, Samantha J. Caesar-Johnson, Andy Cafferkey, Declan Cahill, Claudia Calabrese, Carlos Caldas, Fabien Calvo, Niedzica Camacho, Peter J. Campbell, Elias Campo, Cinzia Cantù, Shaolong Cao, Thomas E. Carey, Joana Carlevaro-Fita, Rebecca Carlsen, Ivana Cataldo, Mario Cazzola, Jonathan Cebon, Robert Cerfolio, Dianne E. Chadwick, Dimple Chakravarty, Don Chalmers, Calvin Wing Yiu Chan, Kin Chan, Michelle Chan-Seng-Yue, Vishal S. Chandan, David K. Chang, Stephen J. Chanock, Lorraine A. Chantrill, Aurélien Chateigner, Nilanjan Chatterjee, Kazuaki Chayama, Hsiao-Wei Chen, Jieming Chen, Ken Chen, Yiwen Chen, Zhaohong Chen, Andrew D. Cherniack, Jeremy Chien, Yoke-Eng Chiew, Suet-Feung Chin, Juok Cho, Sunghoon Cho, Jung Kyoon Choi, Wan Choi, Christine Chomienne, Zechen Chong, Su Pin Choo, Angela Chou, Angelika N. Christ, Elizabeth L. Christie, Eric Chuah, Carrie Cibulskis, Kristian Cibulskis, Sara Cingarlini, Peter Clapham, Alexander Claviez, Sean Cleary, Nicole Cloonan, Marek Cmero, Colin C. Collins, Ashton A. Connor, Susanna L. Cooke, Colin S. Cooper, Leslie Cope, Vincenzo Corbo, Matthew G. Cordes, Stephen M. Cordner, Isidro Cortés-Ciriano, Kyle Covington, Prue A. Cowin, Brian Craft, David Craft, Chad J. Creighton, Yupeng Cun, Erin Curley, Ioana Cutcutache, Karolina Czajka, Bogdan Czerniak, Rebecca A. Dagg, Ludmila Danilova, Maria Vittoria Davi, Natalie R. Davidson, Helen Davies, Ian J. Davis, Brandi N. Davis-Dusenbery, Kevin J. Dawson, Francisco M. De La Vega, Ricardo De Paoli-Iseppi, Timothy Defreitas, Angelo P. Dei Tos, Olivier Delaneau, John A. Demchok, Jonas Demeulemeester, German M. Demidov, Deniz Demircioğlu, Nening M. Dennis, Robert E. Denroche, Stefan C. Dentro, Nikita Desai, Vikram Deshpande, Amit G. Deshwar, Christine Desmedt, Jordi Deu-Pons, Noreen Dhalla, Neesha C. Dhani, Priyanka Dhingra, Rajiv Dhir, Anthony DiBiase, Klev Diamanti, Li Ding, Shuai Ding, Huy Q. Dinh, Luc Dirix, HarshaVardhan Doddapaneni, Nilgun Donmez, Michelle T. Dow, Ronny Drapkin, Oliver Drechsel, Ruben M. Drews, Serge Serge, Tim Dudderidge, Ana Dueso-Barroso, Andrew J. Dunford, Michael Dunn, Lewis Jonathan Dursi, Fraser R. Duthie, Ken Dutton-Regester, Jenna Eagles, Douglas F. Easton, Stuart Edmonds, Paul A. Edwards, Sandra E. Edwards, Rosalind A. Eeles, Anna Ehinger, Juergen Eils, Roland Eils, Adel El-Naggar, Matthew Eldridge, Kyle Ellrott, Serap Erkek, Georgia Escaramis, Shadrielle M. G. Espiritu, Xavier Estivill, Dariush Etemadmoghadam, Jorunn E. Eyfjord, Bishoy M. Faltas, Daiming Fan, Yu Fan, William C. Faquin, Claudiu Farcas, Matteo Fassan, Aquila Fatima, Francesco Favero, Nodirjon Fayzullaev, Ina Felau, Sian Fereday, Martin L. Ferguson, Vincent Ferretti, Lars Feuerbach, Matthew A. Field, J. Lynn Fink, Gaetano Finocchiaro, Cyril Fisher, Matthew W. Fittall, Anna Fitzgerald, Rebecca C. Fitzgerald, Adrienne M. Flanagan, Neil E. Fleshner, Paul Flicek, John A. Foekens, Kwun M. Fong, Nuno A. Fonseca, Christopher S. Foster, Natalie S. Fox, Michael Fraser, Scott Frazer, Milana Frenkel-Morgenstern, William Friedman, Joan Frigola, Catrina C. Fronick, Akihiro Fujimoto, Masashi Fujita, Masashi Fukayama, Lucinda A. Fulton, Robert S. Fulton, Mayuko Furuta, P. Andrew Futreal, Anja Füllgrabe, Stacey B. Gabriel, Steven Gallinger, Carlo Gambacorti-Passerini, Jianjiong Gao, Shengjie Gao, Levi Garraway, Øystein Garred, Erik Garrison, Dale W. Garsed, Nils Gehlenborg, Josep L. L. Gelpi, Joshy George, Daniela S. Gerhard, Clarissa Gerhauser, Jeffrey E. Gershenwald, Mark Gerstein, Moritz Gerstung, Gad Getz, Mohammed Ghori, Ronald Ghossein, Nasra H. Giama, Richard A. Gibbs, Bob Gibson, Anthony J. Gill, Pelvender Gill, Dilip D. Giri, Dominik Glodzik, Vincent J. Gnanapragasam, Maria Elisabeth Goebler, Mary J. Goldman, Carmen Gomez, Santiago Gonzalez, Abel Gonzalez-Perez, Dmitry A. Gordenin, James Gossage, Kunihito Gotoh, Ramaswamy Govindan, Dorthe Grabau, Janet S. Graham, Robert C. Grant, Anthony R. Green, Eric Green, Liliana Greger, Nicola Grehan, Sonia Grimaldi, Sean M. Grimmond, Robert L. Grossman, Adam Grundhoff, Gunes Gundem, Qianyun Guo, Manaswi Gupta, Shailja Gupta, Ivo G. Gut, Marta Gut, Jonathan Göke, Gavin Ha, Andrea Haake, David Haan, Siegfried Haas, Kerstin Haase, James E. Haber, Nina Habermann, Faraz Hach, Syed Haider, Natsuko Hama, Freddie C. Hamdy, Anne Hamilton, Mark P. Hamilton, Leng Han, George B. Hanna, Martin Hansmann, Nicholas J. Haradhvala, Olivier Harismendy, Ivon Harliwong, Arif O. Harmanci, Eoghan Harrington, Takanori Hasegawa, David Haussler, Steve Hawkins, Shinya Hayami, Shuto Hayashi, D. Neil Hayes, Stephen J. Hayes, Nicholas K. Hayward, Steven Hazell, Yao He, Allison P. Heath, Simon C. Heath, David Hedley, Apurva M. Hegde, David I. Heiman, Michael C. Heinold, Zachary Heins, Lawrence E. Heisler, Eva Hellstrom-Lindberg, Mohamed Helmy, Seong Gu Heo, Austin J. Hepperla, José María Heredia-Genestar, Carl Herrmann, Peter Hersey, Julian M. Hess, Holmfridur Hilmarsdottir, Jonathan Hinton, Satoshi Hirano, Nobuyoshi Hiraoka, Katherine A. Hoadley, Asger Hobolth, Ermin Hodzic, Jessica I. Hoell, Steve Hoffmann, Oliver Hofmann, Andrea Holbrook, Aliaksei Z. Holik, Michael A. Hollingsworth, Oliver Holmes, Robert A. Holt, Chen Hong, Eun Pyo Hong, Jongwhi H. Hong, Gerrit K. Hooijer, Henrik Hornshøj, Fumie Hosoda, Yong Hou, Volker Hovestadt, William Howat, Alan P. Hoyle, Ralph H. Hruban, Jianhong Hu, Taobo Hu, Xing Hua, Kuan-lin Huang, Mei Huang, Mi Ni Huang, Vincent Huang, Yi Huang, Wolfgang Huber, Thomas J. Hudson, Michael Hummel, Jillian A. Hung, David Huntsman, Ted R. Hupp, Jason Huse, Matthew R. Huska, Barbara Hutter, Carolyn M. Hutter, Daniel Hübschmann, Christine A. Iacobuzio-Donahue, Charles David Imbusch, Marcin Imielinski, Seiya Imoto, William B. Isaacs, Keren Isaev, Shumpei Ishikawa, Murat Iskar, S. M. Ashiqul Islam, Michael Ittmann, Sinisa Ivkovic, Jose M. G. Izarzugaza, Jocelyne Jacquemier, Valerie Jakrot, Nigel B. Jamieson, Gun Ho Jang, Se Jin Jang, Joy C. Jayaseelan, Reyka Jayasinghe, Stuart R. Jefferys, Karine Jegalian, Jennifer L. Jennings, Seung-Hyup Jeon, Lara Jerman, Yuan Ji, Wei Jiao, Peter A. Johansson, Amber L. Johns, Jeremy Johns, Rory Johnson, Todd A. Johnson, Clemency Jolly, Yann Joly, Jon G. Jonasson, Corbin D. Jones, David R. Jones, David T. W. Jones, Nic Jones, Steven J. M. Jones, Jos Jonkers, Young Seok Ju, Hartmut Juhl, Jongsun Jung, Malene Juul, Randi Istrup Juul, Sissel Juul, Natalie Jäger, Rolf Kabbe, Andre Kahles, Abdullah Kahraman, Vera B. Kaiser, Hojabr Kakavand, Sangeetha Kalimuthu, Christof von Kalle, Koo Jeong Kang, Katalin Karaszi, Beth Karlan, Rosa Karlić, Dennis Karsch, Katayoon Kasaian, Karin S. Kassahn, Hitoshi Katai, Mamoru Kato, Hiroto Katoh, Yoshiiku Kawakami, Jonathan D. Kay, Stephen H. Kazakoff, Marat D. Kazanov, Maria Keays, Electron Kebebew, Richard F. Kefford, Manolis Kellis, James G. Kench, Catherine J. Kennedy, Jules N. A. Kerssemakers, David Khoo, Vincent Khoo, Narong Khuntikeo, Ekta Khurana, Helena Kilpinen, Hark Kyun Kim, Hyung-Lae Kim, Hyung-Yong Kim, Hyunghwan Kim, Jaegil Kim, Jihoon Kim, Jong K. Kim, Youngwook Kim, Tari A. King, Wolfram Klapper, Kortine Kleinheinz, Leszek J. Klimczak, Stian Knappskog, Michael Kneba, Bartha M. Knoppers, Youngil Koh, Jan Komorowski, Daisuke Komura, Mitsuhiro Komura, Gu Kong, Marcel Kool, Jan O. Korbel, Viktoriya Korchina, Andrey Korshunov, Michael Koscher, Roelof Koster, Zsofia Kote-Jarai, Antonios Koures, Milena Kovacevic, Barbara Kremeyer, Helene Kretzmer, Markus Kreuz, Savitri Krishnamurthy, Dieter Kube, Kiran Kumar, Pardeep Kumar, Sushant Kumar, Yogesh Kumar, Ritika Kundra, Kirsten Kübler, Ralf Küppers, Jesper Lagergren, Phillip H. Lai, Peter W. Laird, Sunil R. Lakhani, Christopher M. Lalansingh, Emilie Lalonde, Fabien C. Lamaze, Adam Lambert, Eric Lander, Pablo Landgraf, Luca Landoni, Anita Langerød, Andrés Lanzós, Denis Larsimont, Erik Larsson, Mark Lathrop, Loretta M. S. Lau, Chris Lawerenz, Rita T. Lawlor, Michael S. Lawrence, Alexander J. Lazar, Ana Mijalkovic Lazic, Xuan Le, Darlene Lee, Donghoon Lee, Eunjung Alice Lee, Hee Jin Lee, Jake June-Koo Lee, Jeong-Yeon Lee, Juhee Lee, Ming Ta Michael Lee, Henry Lee-Six, Kjong-Van Lehmann, Hans Lehrach, Dido Lenze, Conrad R. Leonard, Daniel A. Leongamornlert, Ignaty Leshchiner, Louis Letourneau, Ivica Letunic, Douglas A. Levine, Lora Lewis, Tim Ley, Chang Li, Constance H. Li, Haiyan Irene Li, Jun Li, Lin Li, Shantao Li, Siliang Li, Xiaobo Li, Xiaotong Li, Xinyue Li, Yilong Li, Han Liang, Sheng-Ben Liang, Peter Lichter, Pei Lin, Ziao Lin, W. M. Linehan, Ole Christian Lingjærde, Dongbing Liu, Eric Minwei Liu, Fei-Fei Fei Liu, Fenglin Liu, Jia Liu, Xingmin Liu, Julie Livingstone, Dimitri Livitz, Naomi Livni, Lucas Lochovsky, Markus Loeffler, Georgina V. Long, Armando Lopez-Guillermo, Shaoke Lou, David N. Louis, Laurence B. Lovat, Yiling Lu, Yong-Jie Lu, Youyong Lu, Claudio Luchini, Ilinca Lungu, Xuemei Luo, Hayley J. Luxton, Andy G. Lynch, Lisa Lype, Cristina López, Carlos López-Otín, Eric Z. Ma, Yussanne Ma, Gaetan MacGrogan, Shona MacRae, Geoff Macintyre, Tobias Madsen, Kazuhiro Maejima, Andrea Mafficini, Dennis T. Maglinte, Arindam Maitra, Partha P. Majumder, Luca Malcovati, Salem Malikic, Giuseppe Malleo, Graham J. Mann, Luisa Mantovani-Löffler, Kathleen Marchal, Giovanni Marchegiani, Elaine R. Mardis, Adam A. Margolin, Maximillian G. Marin, Florian Markowetz, Julia Markowski, Jeffrey Marks, Tomas Marques-Bonet, Marco A. Marra, Luke Marsden, John W. M. Martens, Sancha Martin, Jose I. Martin-Subero, Iñigo Martincorena, Alexander Martinez-Fundichely, Yosef E. Maruvka, R. Jay Mashl, Charlie E. Massie, Thomas J. Matthew, Lucy Matthews, Erik Mayer, Simon Mayes, Michael Mayo, Faridah Mbabaali, Karen McCune, Ultan McDermott, Patrick D. McGillivray, Michael D. McLellan, John D. McPherson, John R. McPherson, Treasa A. McPherson, Samuel R. Meier, Alice Meng, Shaowu Meng, Andrew Menzies, Neil D. Merrett, Sue Merson, Matthew Meyerson, William Meyerson, Piotr A. Mieczkowski, George L. Mihaiescu, Sanja Mijalkovic, Tom Mikkelsen, Michele Milella, Linda Mileshkin, Christopher A. Miller, David K. Miller, Jessica K. Miller, Gordon B. Mills, Ana Milovanovic, Sarah Minner, Marco Miotto, Gisela Mir Arnau, Lisa Mirabello, Chris Mitchell, Thomas J. Mitchell, Satoru Miyano, Naoki Miyoshi, Shinichi Mizuno, Fruzsina Molnár-Gábor, Malcolm J. Moore, Richard A. Moore, Sandro Morganella, Quaid D. Morris, Carl Morrison, Lisle E. Mose, Catherine D. Moser, Ferran Muiños, Loris Mularoni, Andrew J. Mungall, Karen Mungall, Elizabeth A. Musgrove, Ville Mustonen, David Mutch, Francesc Muyas, Donna M. Muzny, Alfonso Muñoz, Jerome Myers, Ola Myklebost, Peter Möller, Genta Nagae, Adnan M. Nagrial, Hardeep K. Nahal-Bose, Hitoshi Nakagama, Hidewaki Nakagawa, Hiromi Nakamura, Toru Nakamura, Kaoru Nakano, Tannistha Nandi, Jyoti Nangalia, Mia Nastic, Arcadi Navarro, Fabio C. P. Navarro, David E. Neal, Gerd Nettekoven, Felicity Newell, Steven J. Newhouse, Yulia Newton, Alvin Wei Tian Ng, Anthony Ng, Jonathan Nicholson, David Nicol, Yongzhan Nie, G. Petur Nielsen, Morten Muhlig Nielsen, Serena Nik-Zainal, Michael S. Noble, Katia Nones, Paul A. Northcott, Faiyaz Notta, Brian D. O’Connor, Peter O’Donnell, Maria O’Donovan, Sarah O’Meara, Brian Patrick O’Neill, J. Robert O’Neill, David Ocana, Angelica Ochoa, Layla Oesper, Christopher Ogden, Hideki Ohdan, Kazuhiro Ohi, Lucila Ohno-Machado, Karin A. Oien, Akinyemi I. Ojesina, Hidenori Ojima, Takuji Okusaka, Larsson Omberg, Choon Kiat Ong, Stephan Ossowski, German Ott, B. F. Francis Ouellette, Christine P’ng, Marta Paczkowska, Salvatore Paiella, Chawalit Pairojkul, Marina Pajic, Qiang Pan-Hammarström, Elli Papaemmanuil, Irene Papatheodorou, Nagarajan Paramasivam, Ji Wan Park, Joong-Won Park, Keunchil Park, Kiejung Park, Peter J. Park, Joel S. Parker, Simon L. Parsons, Harvey Pass, Danielle Pasternack, Alessandro Pastore, Ann-Marie Patch, Iris Pauporté, Antonio Pea, John V. Pearson, Chandra Sekhar Pedamallu, Jakob Skou Pedersen, Paolo Pederzoli, Martin Peifer, Nathan A. Pennell, Charles M. Perou, Marc D. Perry, Gloria M. Petersen, Myron Peto, Nicholas Petrelli, Robert Petryszak, Stefan M. Pfister, Mark Phillips, Oriol Pich, Hilda A. Pickett, Todd D. Pihl, Nischalan Pillay, Sarah Pinder, Mark Pinese, Andreia V. Pinho, Esa Pitkänen, Xavier Pivot, Elena Piñeiro-Yáñez, Laura Planko, Christoph Plass, Paz Polak, Tirso Pons, Irinel Popescu, Olga Potapova, Aparna Prasad, Shaun R. Preston, Manuel Prinz, Antonia L. Pritchard, Stephenie D. Prokopec, Elena Provenzano, Xose S. Puente, Sonia Puig, Montserrat Puiggròs, Sergio Pulido-Tamayo, Gulietta M. Pupo, Colin A. Purdie, Michael C. Quinn, Raquel Rabionet, Janet S. Rader, Bernhard Radlwimmer, Petar Radovic, Benjamin Raeder, Keiran M. Raine, Manasa Ramakrishna, Kamna Ramakrishnan, Suresh Ramalingam, Benjamin J. Raphael, W. Kimryn Rathmell, Tobias Rausch, Guido Reifenberger, Jüri Reimand, Jorge Reis-Filho, Victor Reuter, Iker Reyes-Salazar, Matthew A. Reyna, Sheila M. Reynolds, Esther Rheinbay, Yasser Riazalhosseini, Andrea L. Richardson, Julia Richter, Matthew Ringel, Markus Ringnér, Yasushi Rino, Karsten Rippe, Jeffrey Roach, Lewis R. Roberts, Nicola D. Roberts, Steven A. Roberts, A. Gordon Robertson, Alan J. Robertson, Javier Bartolomé Rodriguez, Bernardo Rodriguez-Martin, F. Germán Rodríguez-González, Michael H. A. Roehrl, Marius Rohde, Hirofumi Rokutan, Gilles Romieu, Ilse Rooman, Tom Roques, Daniel Rosebrock, Mara Rosenberg, Philip C. Rosenstiel, Andreas Rosenwald, Edward W. Rowe, Romina Royo, Steven G. Rozen, Yulia Rubanova, Mark A. Rubin, Carlota Rubio-Perez, Vasilisa A. Rudneva, Borislav C. Rusev, Andrea Ruzzenente, Gunnar Rätsch, Radhakrishnan Sabarinathan, Veronica Y. Sabelnykova, Sara Sadeghi, S. Cenk Sahinalp, Natalie Saini, Mihoko Saito-Adachi, Gordon Saksena, Adriana Salcedo, Roberto Salgado, Leonidas Salichos, Richard Sallari, Charles Saller, Roberto Salvia, Michelle Sam, Jaswinder S. Samra, Francisco Sanchez-Vega, Chris Sander, Grant Sanders, Rajiv Sarin, Iman Sarrafi, Aya Sasaki-Oku, Torill Sauer, Guido Sauter, Robyn P. M. Saw, Maria Scardoni, Christopher J. Scarlett, Aldo Scarpa, Ghislaine Scelo, Dirk Schadendorf, Jacqueline E. Schein, Markus B. Schilhabel, Matthias Schlesner, Thorsten Schlomm, Heather K. Schmidt, Sarah-Jane Schramm, Stefan Schreiber, Nikolaus Schultz, Steven E. Schumacher, Roland F. Schwarz, Richard A. Scolyer, David Scott, Ralph Scully, Raja Seethala, Ayellet V. Segre, Iris Selander, Colin A. Semple, Yasin Senbabaoglu, Subhajit Sengupta, Elisabetta Sereni, Stefano Serra, Dennis C. Sgroi, Mark Shackleton, Nimish C. Shah, Sagedeh Shahabi, Catherine A. Shang, Ping Shang, Ofer Shapira, Troy Shelton, Ciyue Shen, Hui Shen, Rebecca Shepherd, Ruian Shi, Yan Shi, Yu-Jia Shiah, Tatsuhiro Shibata, Juliann Shih, Eigo Shimizu, Kiyo Shimizu, Seung Jun Shin, Yuichi Shiraishi, Tal Shmaya, Ilya Shmulevich, Solomon I. Shorser, Charles Short, Raunak Shrestha, Suyash S. Shringarpure, Craig Shriver, Shimin Shuai, Nikos Sidiropoulos, Reiner Siebert, Anieta M. Sieuwerts, Lina Sieverling, Sabina Signoretti, Katarzyna O. Sikora, Michele Simbolo, Ronald Simon, Janae V. Simons, Jared T. Simpson, Peter T. Simpson, Samuel Singer, Nasa Sinnott-Armstrong, Payal Sipahimalani, Tara J. Skelly, Marcel Smid, Jaclyn Smith, Karen Smith-McCune, Nicholas D. Socci, Heidi J. Sofia, Matthew G. Soloway, Lei Song, Anil K. Sood, Sharmila Sothi, Christos Sotiriou, Cameron M. Soulette, Paul N. Span, Paul T. Spellman, Nicola Sperandio, Andrew J. Spillane, Oliver Spiro, Jonathan Spring, Johan Staaf, Peter F. Stadler, Peter Staib, Stefan G. Stark, Lucy Stebbings, Ólafur Andri Stefánsson, Oliver Stegle, Lincoln D. Stein, Alasdair Stenhouse, Chip Stewart, Stephan Stilgenbauer, Miranda D. Stobbe, Michael R. Stratton, Jonathan R. Stretch, Adam J. Struck, Joshua M. Stuart, Henk G. Stunnenberg, Hong Su, Xiaoping Su, Ren X. Sun, Stephanie Sungalee, Hana Susak, Akihiro Suzuki, Fred Sweep, Monika Szczepanowski, Holger Sültmann, Takashi Yugawa, Angela Tam, David Tamborero, Benita Kiat Tee Tan, Donghui Tan, Patrick Tan, Hiroko Tanaka, Hirokazu Taniguchi, Tomas J. Tanskanen, Maxime Tarabichi, Roy Tarnuzzer, Patrick Tarpey, Morgan L. Taschuk, Kenji Tatsuno, Simon Tavaré, Darrin F. Taylor, Amaro Taylor-Weiner, Jon W. Teague, Bin Tean Teh, Varsha Tembe, Javier Temes, Kevin Thai, Sarah P. Thayer, Nina Thiessen, Gilles Thomas, Sarah Thomas, Alan Thompson, Alastair M. Thompson, John F. F. Thompson, R. Houston Thompson, Heather Thorne, Leigh B. Thorne, Adrian Thorogood, Grace Tiao, Nebojsa Tijanic, Lee E. Timms, Roberto Tirabosco, Marta Tojo, Stefania Tommasi, Christopher W. Toon, Umut H. Toprak, David Torrents, Giampaolo Tortora, Jörg Tost, Yasushi Totoki, David Townend, Nadia Traficante, Isabelle Treilleux, Jean-Rémi Trotta, Lorenz H. P. Trümper, Ming Tsao, Tatsuhiko Tsunoda, Jose M. C. Tubio, Olga Tucker, Richard Turkington, Daniel J. Turner, Andrew Tutt, Masaki Ueno, Naoto T. Ueno, Christopher Umbricht, Husen M. Umer, Timothy J. Underwood, Lara Urban, Tomoko Urushidate, Tetsuo Ushiku, Liis Uusküla-Reimand, Alfonso Valencia, David J. Van Den Berg, Steven Van Laere, Peter Van Loo, Erwin G. Van Meir, Gert G. Van den Eynden, Theodorus Van der Kwast, Naveen Vasudev, Miguel Vazquez, Ravikiran Vedururu, Umadevi Veluvolu, Shankar Vembu, Lieven P. C. Verbeke, Peter Vermeulen, Clare Verrill, Alain Viari, David Vicente, Caterina Vicentini, K. VijayRaghavan, Juris Viksna, Ricardo E. Vilain, Izar Villasante, Anne Vincent-Salomon, Tapio Visakorpi, Douglas Voet, Paresh Vyas, Ignacio Vázquez-García, Nick M. Waddell, Nicola Waddell, Claes Wadelius, Lina Wadi, Rabea Wagener, Jeremiah A. Wala, Jian Wang, Jiayin Wang, Linghua Wang, Qi Wang, Wenyi Wang, Yumeng Wang, Zhining Wang, Paul M. Waring, Hans-Jörg Warnatz, Jonathan Warrell, Anne Y. Warren, Sebastian M. Waszak, David C. Wedge, Dieter Weichenhan, Paul Weinberger, John N. Weinstein, Joachim Weischenfeldt, Daniel J. Weisenberger, Ian Welch, Michael C. Wendl, Johannes Werner, Justin P. Whalley, David A. Wheeler, Hayley C. Whitaker, Dennis Wigle, Matthew D. Wilkerson, Ashley Williams, James S. Wilmott, Gavin W. Wilson, Julie M. Wilson, Richard K. Wilson, Boris Winterhoff, Jeffrey A. Wintersinger, Maciej Wiznerowicz, Stephan Wolf, Bernice H. Wong, Tina Wong, Winghing Wong, Youngchoon Woo, Scott Wood, Bradly G. Wouters, Adam J. Wright, Derek W. Wright, Mark H. Wright, Chin-Lee Wu, Dai-Ying Wu, Guanming Wu, Jianmin Wu, Kui Wu, Yang Wu, Zhenggang Wu, Liu Xi, Tian Xia, Qian Xiang, Xiao Xiao, Rui Xing, Heng Xiong, Qinying Xu, Yanxun Xu, Hong Xue, Shinichi Yachida, Sergei Yakneen, Rui Yamaguchi, Takafumi N. Yamaguchi, Masakazu Yamamoto, Shogo Yamamoto, Hiroki Yamaue, Fan Yang, Huanming Yang, Jean Y. Yang, Liming Yang, Lixing Yang, Shanlin Yang, Tsun-Po Yang, Yang Yang, Xiaotong Yao, Marie-Laure Yaspo, Lucy Yates, Christina Yau, Chen Ye, Kai Ye, Venkata D. Yellapantula, Christopher J. Yoon, Sung-Soo Yoon, Fouad Yousif, Jun Yu, Kaixian Yu, Willie Yu, Yingyan Yu, Ke Yuan, Yuan Yuan, Denis Yuen, Christina K. Yung, Olga Zaikova, Jorge Zamora, Marc Zapatka, Jean C. Zenklusen, Thorsten Zenz, Nikolajs Zeps, Cheng-Zhong Zhang, Fan Zhang, Hailei Zhang, Hongwei Zhang, Hongxin Zhang, Jiashan Zhang, Jing Zhang, Junjun Zhang, Xiuqing Zhang, Xuanping Zhang, Yan Zhang, Zemin Zhang, Zhongming Zhao, Liangtao Zheng, Xiuqing Zheng, Wanding Zhou, Yong Zhou, Bin Zhu, Hongtu Zhu, Jingchun Zhu, Shida Zhu, Lihua Zou, Xueqing Zou, Anna deFazio, Nicholas van As, Carolien H. M. van Deurzen, Marc J. van de Vijver, L. van’t Veer, Christian von Mering

**Affiliations:** 1 Division of Applied Bioinformatics, German Cancer Research Center (DKFZ), 69120 Heidelberg, Germany; 2 Faculty of Biosciences, Heidelberg University, 69120 Heidelberg, Germany; 3 Division of Theoretical Bioinformatics, German Cancer Research Center (DKFZ), 69120 Heidelberg, Germany; 4 Department for Bioinformatics and Functional Genomics, Institute for Pharmacy and Molecular Biotechnology (IPMB) and BioQuant, 69120 Heidelberg, Germany; 5 German Cancer Consortium (DKTK), Heidelberg, Germany; 6 National Center for Tumor Diseases (NCT) Heidelberg, Heidelberg, Germany; 7 Heidelberg Center for Personalized Oncology (DKFZ-HIPO), German Cancer Research Center (DKFZ), Heidelberg, Germany; 8 Division of Chromatin Networks, German Cancer Research Center (DKFZ) and BioQuant, 69120 Heidelberg, Germany; 9 Department of Biomedical Informatics, Harvard Medical School, Boston, Massachusetts 02115 USA; 10 Department of Chemistry, Centre for Molecular Science Informatics, University of Cambridge, Cambridge, CB2 1EW UK; 11 Ludwig Center at Harvard Medical School, Boston, Massachusetts 02115 USA; 12 School of Mathematical Sciences and Center for Statistical Science, Peking University, Beijing, 100871 China; 13 Bioinformatics and Omics Data Analytics, German Cancer Research Center (DKFZ), 69120 Heidelberg, Germany; 14 Hopp Children’s Cancer Center (KiTZ), Heidelberg, Germany; 15 Pediatric Glioma Research Group, German Cancer Research Center (DKFZ), Heidelberg, Germany; 16 University of Texas MD Anderson Cancer Center, Houston, TX 77030 USA; 17 Department of Zoology, Genetics and Physical Anthropology, Universidade de Santiago de Compostela, Santiago de Compostela, 15706 Spain; 18 Centre for Research in Molecular Medicine and Chronic Diseases (CIMUS), Universidade de Santiago de Compostela, Santiago de Compostela, 15706 Spain; 19 The Biomedical Research Centre (CINBIO), Universidade de Vigo, Vigo, 36310 Spain; 20 Transmissible Cancer Group, Department of Veterinary Medicine, University of Cambridge, Cambridge, CB3 0ES UK; 21 Broad Institute of MIT and Harvard, Cambridge, MA 02142 USA; 22 Department of Medical Oncology, Dana-Farber Cancer Institute, Boston, MA 02115 USA; 23 Harvard Medical School, Boston, MA 02115 USA; 24 Computational Biology Program, Ontario Institute for Cancer Research, Toronto, ON M5G 0A3 Canada; 25 Department of Medical Biophysics, University of Toronto, Toronto, ON M5S 1A8 Canada; 26 Department of Pharmacology, University of Toronto, Toronto, ON M5S 1A8 Canada; 27 University of California Los Angeles, Los Angeles, CA 90095 USA; 28 Peter MacCallum Cancer Centre, Melbourne, VIC 3000 Australia; 29 Sir Peter MacCallum Department of Oncology, University of Melbourne, Melbourne, VIC 3052 Australia; 30 Johns Hopkins School of Medicine, Baltimore, MD 21205 USA; 31 Wellcome Sanger Institute, Wellcome Genome Campus, Hinxton, Cambridge CB10 1SA UK; 32 Department of Haematology, University of Cambridge, Cambridge, CB2 2XY UK; 33 University of Ottawa Faculty of Medicine, Department of Biochemistry, Microbiology and Immunology, Ottawa, ON K1H 8M5 Canada; 34 Barcelona Supercomputing Center (BSC), Barcelona, 08034 Spain; 35 Cancer Research UK Cambridge Institute, University of Cambridge, Cambridge, CB2 0RE UK; 36 University of Cambridge, Cambridge, CB2 1TN UK; 37 Quantitative Genomics Laboratories (qGenomics), Barcelona, 08950 Spain; 38 Centre for Genomic Regulation (CRG), The Barcelona Institute of Science and Technology, Barcelona, 08003 Spain; 39 Queensland Centre for Medical Genomics, Institute for Molecular Bioscience, The University of Queensland, St Lucia, QLD 4072 Australia; 40 The Azrieli Faculty of Medicine, Bar-Ilan University, Safed, 13195 Israel; 41 Department of Computer Science, Yale University, New Haven, CT 06520 USA; 42 Department of Molecular Biophysics and Biochemistry, Yale University, New Haven, CT 06520 USA; 43 Program in Computational Biology and Bioinformatics, Yale University, New Haven, CT 06520 USA; 44 Genome Integrity and Structural Biology Laboratory, National Institute of Environmental Health Sciences (NIEHS), Durham, NC 27709 USA; 45 Biomolecular Engineering Department, University of California, Santa Cruz, Santa Cruz, CA 95064 USA; 46 Brandeis University, Waltham, MA 02254 USA; 47 Massachusetts General Hospital Center for Cancer Research, Charlestown, MA 02129 USA; 48 New York Genome Center, New York, NY 10013 USA; 49 Weill Cornell Medicine, New York, NY 10065 USA; 50 Korea Advanced Institute of Science and Technology, Daejeon, 34141 South Korea; 51 Skolkovo Institute of Science and Technology, Moscow, 121205 Russia; 52 A.A.Kharkevich Institute of Information Transmission Problems, Moscow, 127051 Russia; 53 Dmitry Rogachev National Research Center of Pediatric Hematology, Oncology and Immunology, Moscow, 117997 Russia; 54 Integrative Bioinformatics Support Group, National Institute of Environmental Health Sciences (NIEHS), Durham, NC 27709 USA; 55 Center For Medical Innovation, Seoul National University Hospital, Seoul, 03080 South Korea; 56 Department of Internal Medicine, Seoul National University Hospital, Seoul, 03080 South Korea; 57 European Molecular Biology Laboratory, European Bioinformatics Institute (EMBL-EBI), Wellcome Genome Campus, Hinxton, Cambridge, CB10 1SD UK; 58 Genome Biology Unit, European Molecular Biology Laboratory (EMBL), Heidelberg, 69117 Germany; 59 Division of Genetics and Genomics, Boston Children’s Hospital and Harvard Medical School, Boston, MA 02115 USA; 60 School of Medicine/School of Mathematics and Statistics, University of St Andrews, St Andrews, Fife, KY16 9SS UK; 61 Department of Physiology and Biophysics, Weill Cornell Medicine, New York, NY 10065 USA; 62 Englander Institute for Precision Medicine, Weill Cornell Medicine, New York, NY 10065 USA; 63 Institute for Computational Biomedicine, Weill Cornell Medicine, New York, NY 10021 USA; 64 Dana-Farber Cancer Institute, Boston, MA 02215 USA; 65 Department of Medical Oncology, Inselspital, University Hospital and University of Bern, Bern, 3010 Switzerland; 66 Department of Pathology, The University of Melbourne, Melbourne, VIC 3052 Australia; 67 The Institute of Medical Science, The University of Tokyo, Tokyo, 108-8639 Japan; 68 RIKEN Center for Integrative Medical Sciences, Yokohama, Kanagawa 230-0045 Japan; 69 Institute of Medical Genetics and Applied Genomics, University of Tübingen, Tübingen, 72074 Germany; 70 Universitat Pompeu Fabra (UPF), Barcelona, 08003 Spain; 71 Department of Genetics and Computational Biology, QIMR Berghofer Medical Research Institute, Brisbane, 4006 Australia; 72 Institute for Molecular Bioscience, University of Queensland, St Lucia, Brisbane, QLD 4072 Australia; 73 School of Molecular Biosciences and Center for Reproductive Biology, Washington State University, Pullman, WA 99164 USA; 74 Department of Cancer Biology, Dana-Farber Cancer Institute, Boston, MA 02215 USA; 75 Cancer Research Institute, Beth Israel Deaconess Medical Center, Boston, MA 02215 USA; 76 Peter MacCallum Cancer Centre and University of Melbourne, Melbourne, VIC 3000 Australia; 77 Finsen Laboratory and Biotech Research & Innovation Centre (BRIC), University of Copenhagen, Copenhagen, 2200 Denmark; 78 Institució Catalana de Recerca i Estudis Avançats (ICREA), Barcelona, 08010 Spain; 79 Department of Urology, Charité Universitätsmedizin Berlin, Berlin, 10117 Germany; 80 Ben May Department for Cancer Research, Department of Human Genetics, The University of Chicago, Chicago, IL 60637 USA; 81 Tri-institutional PhD program of computational biology and medicine, Weill Cornell Medicine, New York, NY 10065 USA; 200 Applied Tumor Genomics Research Program, Research Programs Unit, University of Helsinki, Helsinki, Finland; 201 Wellcome Sanger Institute, Wellcome Genome Campus, Hinxton, UK; 202 Memorial Sloan Kettering Cancer Center, New York, NY USA; 203 Genome Science Division, Research Center for Advanced Science and Technology, University of Tokyo, Tokyo, Japan; 204 Department of Surgery, University of Chicago, Chicago, IL USA; 205 Department of Surgery, Division of Hepatobiliary and Pancreatic Surgery, School of Medicine, Keimyung University Dongsan Medical Center, Daegu, South Korea; 206 Department of Oncology, Gil Medical Center, Gachon University, Incheon, South Korea; 207 Hiroshima University, Hiroshima, Japan; 208 Department of Bioinformatics and Computational Biology, The University of Texas MD Anderson Cancer Center, Houston, TX USA; 209 University of Texas MD Anderson Cancer Center, Houston, TX USA; 210 King Faisal Specialist Hospital and Research Centre, Al Maather, Riyadh, Saudi Arabia; 211 Bioinformatics Unit, Spanish National Cancer Research Centre (CNIO), Madrid, Spain; 212 Bioinformatics Core Facility, University Medical Center Hamburg, Hamburg, Germany; 213 Heinrich Pette Institute, Leibniz Institute for Experimental Virology, Hamburg, Germany; 214 Ontario Tumour Bank, Ontario Institute for Cancer Research, Toronto, ON Canada; 215 Department of Pathology, The University of Texas MD Anderson Cancer Center, Houston, TX USA; 216 Laboratory of Pathology, Center for Cancer Research, National Cancer Institute, Bethesda, MD USA; 217 Department of Cellular and Molecular Medicine and Department of Bioengineering, University of California San Diego, La Jolla, CA USA; 218 UC San Diego Moores Cancer Center, San Diego, CA USA; 219 Canada’s Michael Smith Genome Sciences Centre, BC Cancer, Vancouver, BC Canada; 220 Sir Peter MacCallum Department of Oncology, Peter MacCallum Cancer Centre, University of Melbourne, Melbourne, VIC Australia; 221 Centre for Research in Molecular Medicine and Chronic Diseases (CiMUS), Universidade de Santiago de Compostela, Santiago de Compostela, Spain; 222 Department of Zoology, Genetics and Physical Anthropology, (CiMUS), Universidade de Santiago de Compostela, Santiago de Compostela, Spain; 223 The Biomedical Research Centre (CINBIO), Universidade de Vigo, Vigo, Spain; 224 Royal National Orthopaedic Hospital - Bolsover, London, UK; 225 Department of Genomic Medicine, The University of Texas MD Anderson Cancer Center, Houston, TX USA; 226 Quantitative and Computational Biosciences Graduate Program, Baylor College of Medicine, Houston, TX USA; 227 The Jackson Laboratory for Genomic Medicine, Farmington, CT USA; 228 Genome Informatics Program, Ontario Institute for Cancer Research, Toronto, ON Canada; 229 Institute of Human Genetics, Christian-Albrechts-University, Kiel, Germany; 230 Institute of Human Genetics, Ulm University and Ulm University Medical Center, Ulm, Germany; 231 Queensland Centre for Medical Genomics, Institute for Molecular Bioscience, University of Queensland, St. Lucia, Brisbane, QLD Australia; 232 Salford Royal NHS Foundation Trust, Salford, UK; 233 Department of Surgery, Pancreas Institute, University and Hospital Trust of Verona, Verona, Italy; 234 Molecular and Medical Genetics, OHSU Knight Cancer Institute, Oregon Health and Science University, Portland, OR USA; 235 Department of Molecular Oncology, BC Cancer Research Centre, Vancouver, BC Canada; 236 The McDonnell Genome Institute at Washington University, St. Louis, MO USA; 237 University College London, London, UK; 238 Division of Cancer Genomics, National Cancer Center Research Institute, National Cancer Center, Tokyo, Japan; 239 DLR Project Management Agency, Bonn, Germany; 240 Tokyo Women’s Medical University, Tokyo, Japan; 241 Center for Molecular Oncology, Memorial Sloan Kettering Cancer Center, New York, NY USA; 242 Los Alamos National Laboratory, Los Alamos, NM USA; 243 Department of Pathology, University Health Network, Toronto General Hospital, Toronto, ON Canada; 244 Nottingham University Hospitals NHS Trust, Nottingham, UK; 245 Epigenomics and Cancer Risk Factors, German Cancer Research Center (DKFZ), Heidelberg, Germany; 246 Computational Biology Program, Ontario Institute for Cancer Research, Toronto, ON Canada; 247 Department of Molecular Genetics, University of Toronto, Toronto, ON Canada; 248 Vector Institute, Toronto, ON Canada; 249 Hematopathology Section, Institute of Pathology, Christian-Albrechts-University, Kiel, Germany; 250 Department of Pathology and Laboratory Medicine, School of Medicine, University of North Carolina at Chapel Hill, Chapel Hill, NC USA; 251 Department of Cancer Genetics, Institute for Cancer Research, Oslo University Hospital, The Norwegian Radium Hospital, Oslo, Norway; 252 Pathology, Hospital Clinic, Institut d’Investigacions Biomèdiques August Pi i Sunyer (IDIBAPS), University of Barcelona, Barcelona, Spain; 253 Department of Veterinary Medicine, Transmissible Cancer Group, University of Cambridge, Cambridge, UK; 254 Alvin J. Siteman Cancer Center, Washington University School of Medicine, St. Louis, MO USA; 255 Wolfson Wohl Cancer Research Centre, Institute of Cancer Sciences, University of Glasgow, Glasgow, UK; 256 Lineberger Comprehensive Cancer Center, University of North Carolina at Chapel Hill, Chapel Hill, NC USA; 257 Broad Institute of MIT and Harvard, Cambridge, MA USA; 258 Dana-Farber/Boston Children’s Cancer and Blood Disorders Center, Boston, MA USA; 259 Department of Pediatrics, Harvard Medical School, Boston, MA USA; 260 Leeds Institute of Medical Research @ St. James’s, University of Leeds, St. James’s University Hospital, Leeds, UK; 261 Department of Pathology and Diagnostics, University and Hospital Trust of Verona, Verona, Italy; 262 Department of Surgery, Princess Alexandra Hospital, Brisbane, QLD Australia; 263 Surgical Oncology Group, Diamantina Institute, University of Queensland, Brisbane, QLD Australia; 264 Department of Population and Quantitative Health Sciences, Case Western Reserve University School of Medicine, Cleveland, OH USA; 265 Research Health Analytics and Informatics, University Hospitals Cleveland Medical Center, Cleveland, OH USA; 266 Gloucester Royal Hospital, Gloucester, UK; 267 European Molecular Biology Laboratory, European Bioinformatics Institute (EMBL-EBI), Cambridge, UK; 268 Diagnostic Development, Ontario Institute for Cancer Research, Toronto, ON Canada; 269 Barcelona Supercomputing Center (BSC), Barcelona, Spain; 270 Arnie Charbonneau Cancer Institute, University of Calgary, Calgary, AB Canada; 271 Departments of Surgery and Oncology, University of Calgary, Calgary, AB Canada; 272 Department of Pathology, Oslo University Hospital, The Norwegian Radium Hospital, Oslo, Norway; 273 PanCuRx Translational Research Initiative, Ontario Institute for Cancer Research, Toronto, ON Canada; 274 Department of Oncology, Sidney Kimmel Comprehensive Cancer Center at Johns Hopkins University School of Medicine, Baltimore, MD USA; 275 University Hospital Southampton NHS Foundation Trust, Southampton, UK; 276 Royal Stoke University Hospital, Stoke-on-Trent, UK; 277 Genome Sequence Informatics, Ontario Institute for Cancer Research, Toronto, ON Canada; 278 Human Longevity Inc, San Diego, CA USA; 279 Olivia Newton-John Cancer Research Institute, La Trobe University, Heidelberg, VIC Australia; 280 Computer Network Information Center, Chinese Academy of Sciences, Beijing, China; 281 Genome Canada, Ottawa, ON Canada; 282 CNAG-CRG, Centre for Genomic Regulation (CRG), Barcelona Institute of Science and Technology (BIST), Barcelona, Spain; 283 Universitat Pompeu Fabra (UPF), Barcelona, Spain; 284 Buck Institute for Research on Aging, Novato, CA USA; 285 Duke University Medical Center, Durham, NC USA; 286 Department of Human Genetics, Hannover Medical School, Hannover, Germany; 287 Center for Bioinformatics and Functional Genomics, Cedars-Sinai Medical Center, Los Angeles, CA USA; 288 Department of Biomedical Sciences, Cedars-Sinai Medical Center, Los Angeles, CA USA; 289 The Hebrew University Faculty of Medicine, Jerusalem, Israel; 290 Barts Cancer Institute, Barts and the London School of Medicine and Dentistry, Queen Mary University of London, London, UK; 291 Department of Computer Science, Bioinformatics Group, University of Leipzig, Leipzig, Germany; 292 Interdisciplinary Center for Bioinformatics, University of Leipzig, Leipzig, Germany; 293 Transcriptome Bioinformatics, LIFE Research Center for Civilization Diseases, University of Leipzig, Leipzig, Germany; 294 Department of Medical Oncology, Dana-Farber Cancer Institute, Boston, MA USA; 295 Department of Cancer Biology, Dana-Farber Cancer Institute, Boston, MA USA; 296 Harvard Medical School, Boston, MA USA; 297 USC Norris Comprehensive Cancer Center, University of Southern California, Los Angeles, CA USA; 298 Department of Diagnostics and Public Health, University and Hospital Trust of Verona, Verona, Italy; 299 Department of Mathematics, Aarhus University, Aarhus, Denmark; 300 Department of Molecular Medicine (MOMA), Aarhus University Hospital, Aarhus N, Denmark; 301 Instituto Carlos Slim de la Salud, Mexico City, Mexico; 302 Department of Medical Biophysics, University of Toronto, Toronto, ON Canada; 303 Cancer Division, Garvan Institute of Medical Research, Kinghorn Cancer Centre, University of New South Wales (UNSW Sydney), Sydney, NSW Australia; 304 South Western Sydney Clinical School, Faculty of Medicine, University of New South Wales (UNSW Sydney), Liverpool, NSW Australia; 305 West of Scotland Pancreatic Unit, Glasgow Royal Infirmary, Glasgow, UK; 306 Center for Digital Health, Berlin Institute of Health and Charitè - Universitätsmedizin Berlin, Berlin, Germany; 307 Heidelberg Center for Personalized Oncology (DKFZ-HIPO), German Cancer Research Center (DKFZ), Heidelberg, Germany; 308 The Preston Robert Tisch Brain Tumor Center, Duke University Medical Center, Durham, NC USA; 309 Massachusetts General Hospital, Boston, MA USA; 310 National Institute of Biomedical Genomics, Kalyani, West Bengal India; 311 Institute of Clinical Medicine and Institute of Oral Biology, University of Oslo, Oslo, Norway; 312 University of North Carolina at Chapel Hill, Chapel Hill, NC USA; 313 ARC-Net Centre for Applied Research on Cancer, University and Hospital Trust of Verona, Verona, Italy; 314 The Institute of Cancer Research, London, UK; 315 Centre for Computational Biology, Duke-NUS Medical School, Singapore, Singapore; 316 Programme in Cancer and Stem Cell Biology, Duke-NUS Medical School, Singapore, Singapore; 317 Division of Oncology and Pathology, Department of Clinical Sciences Lund, Lund University, Lund, Sweden; 318 Department of Pediatric Oncology, Hematology and Clinical Immunology, Heinrich-Heine-University, Düsseldorf, Germany; 319 Laboratory for Medical Science Mathematics, RIKEN Center for Integrative Medical Sciences, Yokohama, Japan; 320 RIKEN Center for Integrative Medical Sciences, Yokohama, Japan; 321 Department of Internal Medicine/Hematology, Friedrich-Ebert-Hospital, Neumünster, Germany; 322 Departments of Dermatology and Pathology, Yale University, New Haven, CT USA; 323 Centre for Genomic Regulation (CRG), The Barcelona Institute of Science and Technology, Barcelona, Spain; 324 Radcliffe Department of Medicine, University of Oxford, Oxford, UK; 325 Canadian Center for Computational Genomics, McGill University, Montreal, QC Canada; 326 Department of Human Genetics, McGill University, Montreal, QC Canada; 327 Department of Human Genetics, University of California Los Angeles, Los Angeles, CA USA; 328 Department of Pharmacology, University of Toronto, Toronto, ON Canada; 329 Faculty of Medicine and Health Technology, Tampere University and Tays Cancer Center, Tampere University Hospital, Tampere, Finland; 330 Haematology, Leeds Teaching Hospitals NHS Trust, Leeds, UK; 331 Translational Research and Innovation, Centre Léon Bérard, Lyon, France; 332 Fox Chase Cancer Center, Philadelphia, PA USA; 333 International Agency for Research on Cancer, World Health Organization, Lyon, France; 334 Earlham Institute, Norwich, UK; 335 Norwich Medical School, University of East Anglia, Norwich, UK; 336 Department of Molecular Biology, Faculty of Science, Radboud Institute for Molecular Life Sciences, Radboud University, Nijmegen, HB The Netherlands; 337 CRUK Manchester Institute and Centre, Manchester, UK; 338 Department of Radiation Oncology, University of Toronto, Toronto, ON Canada; 339 Division of Cancer Sciences, Manchester Cancer Research Centre, University of Manchester, Manchester, UK; 340 Radiation Medicine Program, Princess Margaret Cancer Centre, Toronto, ON Canada; 341 Department of Pathology, Brigham and Women’s Hospital, Harvard Medical School, Boston, MA USA; 342 Department of Surgery, Division of Thoracic Surgery, The Johns Hopkins University School of Medicine, Baltimore, MD USA; 343 Division of Molecular Pathology, The Netherlands Cancer Institute, Oncode Institute, Amsterdam, CX The Netherlands; 344 Department of Biomolecular Engineering, University of California Santa Cruz, Santa Cruz, CA USA; 345 UC Santa Cruz Genomics Institute, University of California Santa Cruz, Santa Cruz, CA USA; 346 Division of Applied Bioinformatics, German Cancer Research Center (DKFZ), Heidelberg, Germany; 347 German Cancer Consortium (DKTK), German Cancer Research Center (DKFZ), Heidelberg, Germany; 348 National Center for Tumor Diseases (NCT) Heidelberg, Heidelberg, Germany; 349 Center for Biological Sequence Analysis, Department of Bio and Health Informatics, Technical University of Denmark, Lyngby, Denmark; 350 Novo Nordisk Foundation Center for Protein Research, University of Copenhagen, Copenhagen, Denmark; 351 Institute for Molecular Bioscience, University of Queensland, St. Lucia, Brisbane, QLD Australia; 352 Biomedical Engineering, Oregon Health and Science University, Portland, OR USA; 353 Division of Theoretical Bioinformatics, German Cancer Research Center (DKFZ), Heidelberg, Germany; 354 Institute of Pharmacy and Molecular Biotechnology and BioQuant, Heidelberg University, Heidelberg, Germany; 355 Federal Ministry of Education and Research, Berlin, Germany; 356 Melanoma Institute Australia, University of Sydney, Sydney, NSW Australia; 357 Pediatric Hematology and Oncology, University Hospital Muenster, Muenster, Germany; 358 Department of Pathology, Johns Hopkins University School of Medicine, Baltimore, MD USA; 359 McKusick-Nathans Institute of Genetic Medicine, Sidney Kimmel Comprehensive Cancer Center at Johns Hopkins University School of Medicine, Baltimore, MD USA; 360 Foundation Medicine, Inc, Cambridge, MA USA; 361 Department of Biomedical Data Science, Stanford University School of Medicine, Stanford, CA USA; 362 Department of Genetics, Stanford University School of Medicine, Stanford, CA USA; 363 Bakar Computational Health Sciences Institute and Department of Pediatrics, University of California, San Francisco, CA USA; 364 Institute of Clinical Medicine, Faculty of Medicine, University of Oslo, Oslo, Norway; 365 National Cancer Institute, National Institutes of Health, Bethesda, MD USA; 366 Royal Marsden NHS Foundation Trust, London and Sutton, UK; 367 Genome Biology Unit, European Molecular Biology Laboratory (EMBL), Heidelberg, Germany; 368 Department of Oncology, University of Cambridge, Cambridge, UK; 369 Li Ka Shing Centre, Cancer Research UK Cambridge Institute, University of Cambridge, Cambridge, UK; 370 Institut Gustave Roussy, Villejuif, France; 371 Cambridge University Hospitals NHS Foundation Trust, Cambridge, UK; 372 Department of Haematology, University of Cambridge, Cambridge, UK; 373 Anatomia Patológica, Hospital Clinic, Institut d’Investigacions Biomèdiques August Pi i Sunyer (IDIBAPS), University of Barcelona, Barcelona, Spain; 374 Spanish Ministry of Science and Innovation, Madrid, Spain; 375 University of Michigan Comprehensive Cancer Center, Ann Arbor, MI USA; 376 Department for BioMedical Research, University of Bern, Bern, Switzerland; 377 Department of Medical Oncology, Inselspital, University Hospital and University of Bern, Bern, Switzerland; 378 Graduate School for Cellular and Biomedical Sciences, University of Bern, Bern, Switzerland; 379 University of Pavia, Pavia, Italy; 380 University of Alabama at Birmingham, Birmingham, AL USA; 381 UHN Program in BioSpecimen Sciences, Toronto General Hospital, Toronto, ON Canada; 382 Department of Urology, Icahn School of Medicine at Mount Sinai, New York, NY USA; 383 Centre for Law and Genetics, University of Tasmania, Sandy Bay Campus, Hobart, TAS Australia; 384 Faculty of Biosciences, Heidelberg University, Heidelberg, Germany; 385 Department of Biochemistry, Microbiology and Immunology, Faculty of Medicine, University of Ottawa, Ottawa, ON Canada; 386 Division of Anatomic Pathology, Mayo Clinic, Rochester, MN USA; 387 Division of Cancer Epidemiology and Genetics, National Cancer Institute, National Institutes of Health, Bethesda, MD USA; 388 Illawarra Shoalhaven Local Health District L3 Illawarra Cancer Care Centre, Wollongong Hospital, Wollongong, NSW Australia; 389 BioForA, French National Institute for Agriculture, Food, and Environment (INRAE), ONF, Orléans, France; 390 Department of Biostatistics, Bloomberg School of Public Health, Johns Hopkins University, Baltimore, MD USA; 391 University of California San Diego, San Diego, CA USA; 392 Division of Experimental Pathology, Mayo Clinic, Rochester, MN USA; 393 Centre for Cancer Research, The Westmead Institute for Medical Research, University of Sydney, Sydney, NSW Australia; 394 Department of Gynaecological Oncology, Westmead Hospital, Sydney, NSW Australia; 395 PDXen Biosystems Inc, Seoul, South Korea; 396 Korea Advanced Institute of Science and Technology, Daejeon, South Korea; 397 Electronics and Telecommunications Research Institute, Daejeon, South Korea; 398 Institut National du Cancer (INCA), Boulogne-Billancourt, France; 399 Department of Genetics, Informatics Institute, University of Alabama at Birmingham, Birmingham, AL USA; 400 Division of Medical Oncology, National Cancer Centre, Singapore, Singapore; 401 Medical Oncology, University and Hospital Trust of Verona, Verona, Italy; 402 Department of Pediatrics, University Hospital Schleswig-Holstein, Kiel, Germany; 403 Hepatobiliary/Pancreatic Surgical Oncology Program, University Health Network, Toronto, ON Canada; 404 School of Biological Sciences, University of Auckland, Auckland, New Zealand; 405 Department of Surgery, University of Melbourne, Parkville, VIC Australia; 406 The Murdoch Children’s Research Institute, Royal Children’s Hospital, Parkville, VIC Australia; 407 Walter and Eliza Hall Institute, Parkville, VIC Australia; 408 Vancouver Prostate Centre, Vancouver, Canada; 409 Lunenfeld-Tanenbaum Research Institute, Mount Sinai Hospital, Toronto, ON Canada; 410 University of East Anglia, Norwich, UK; 411 Norfolk and Norwich University Hospital NHS Trust, Norwich, UK; 412 Victorian Institute of Forensic Medicine, Southbank, VIC Australia; 413 Department of Biomedical Informatics, Harvard Medical School, Boston, MA USA; 414 Department of Chemistry, Centre for Molecular Science Informatics, University of Cambridge, Cambridge, UK; 415 Ludwig Center at Harvard Medical School, Boston, MA USA; 416 Human Genome Sequencing Center, Baylor College of Medicine, Houston, TX USA; 417 Peter MacCallum Cancer Centre, University of Melbourne, Melbourne, VIC Australia; 418 Physics Division, Optimization and Systems Biology Lab, Massachusetts General Hospital, Boston, MA USA; 419 Department of Medicine, Baylor College of Medicine, Houston, TX USA; 420 University of Cologne, Cologne, Germany; 421 International Genomics Consortium, Phoenix, AZ USA; 422 Genomics Research Program, Ontario Institute for Cancer Research, Toronto, ON Canada; 423 Barking Havering and Redbridge University Hospitals NHS Trust, Romford, UK; 424 Children’s Hospital at Westmead, University of Sydney, Sydney, NSW Australia; 425 Department of Medicine, Section of Endocrinology, University and Hospital Trust of Verona, Verona, Italy; 426 Computational Biology Center, Memorial Sloan Kettering Cancer Center, New York, NY USA; 427 Department of Biology, ETH Zurich, Zürich, Switzerland; 428 Department of Computer Science, ETH Zurich, Zurich, Switzerland; 429 SIB Swiss Institute of Bioinformatics, Lausanne, Switzerland; 430 Weill Cornell Medical College, New York, NY USA; 431 Academic Department of Medical Genetics, University of Cambridge, Addenbrooke’s Hospital, Cambridge, UK; 432 MRC Cancer Unit, University of Cambridge, Cambridge, UK; 433 Departments of Pediatrics and Genetics, University of North Carolina at Chapel Hill, Chapel Hill, NC USA; 434 Seven Bridges Genomics, Charlestown, MA USA; 435 Annai Systems, Inc, Carlsbad, CA USA; 436 Department of Pathology, General Hospital of Treviso, Department of Medicine, University of Padua, Treviso, Italy; 437 Department of Computational Biology, University of Lausanne, Lausanne, Switzerland; 438 Department of Genetic Medicine and Development, University of Geneva Medical School, Geneva, CH Switzerland; 439 Swiss Institute of Bioinformatics, University of Geneva, Geneva, CH Switzerland; 440 The Francis Crick Institute, London, UK; 441 University of Leuven, Leuven, Belgium; 442 Institute of Medical Genetics and Applied Genomics, University of Tübingen, Tübingen, Germany; 443 Computational and Systems Biology, Genome Institute of Singapore, Singapore, Singapore; 444 School of Computing, National University of Singapore, Singapore, Singapore; 445 Big Data Institute, Li Ka Shing Centre, University of Oxford, Oxford, UK; 446 Biomedical Data Science Laboratory, Francis Crick Institute, London, UK; 447 Bioinformatics Group, Department of Computer Science, University College London, London, UK; 448 The Edward S. Rogers Sr. Department of Electrical and Computer Engineering, University of Toronto, Toronto, ON Canada; 449 Breast Cancer Translational Research Laboratory JC Heuson, Institut Jules Bordet, Brussels, Belgium; 450 Department of Oncology, Laboratory for Translational Breast Cancer Research, KU Leuven, Leuven, Belgium; 451 Institute for Research in Biomedicine (IRB Barcelona), The Barcelona Institute of Science and Technology, Barcelona, Spain; 452 Research Program on Biomedical Informatics, Universitat Pompeu Fabra, Barcelona, Spain; 453 Division of Medical Oncology, Princess Margaret Cancer Centre, Toronto, ON Canada; 454 Department of Physiology and Biophysics, Weill Cornell Medicine, New York, NY USA; 455 Institute for Computational Biomedicine, Weill Cornell Medicine, New York, NY USA; 456 Department of Pathology, UPMC Shadyside, Pittsburgh, PA USA; 457 Independent Consultant, Wellesley, USA; 458 Department of Cell and Molecular Biology, Science for Life Laboratory, Uppsala University, Uppsala, Sweden; 459 Department of Medicine and Department of Genetics, Washington University School of Medicine, St. Louis, St. Louis, MO USA; 460 Hefei University of Technology, Anhui, China; 461 Translational Cancer Research Unit, GZA Hospitals St.-Augustinus, Center for Oncological Research, Faculty of Medicine and Health Sciences, University of Antwerp, Antwerp, Belgium; 462 Simon Fraser University, Burnaby, BC Canada; 463 University of Pennsylvania, Philadelphia, PA USA; 464 Faculty of Science and Technology, University of Vic—Central University of Catalonia (UVic-UCC), Vic, Spain; 465 The Wellcome Trust, London, UK; 466 The Hospital for Sick Children, Toronto, ON Canada; 467 Department of Pathology, Queen Elizabeth University Hospital, Glasgow, UK; 468 Department of Genetics and Computational Biology, QIMR Berghofer Medical Research Institute, Brisbane, QLD Australia; 469 Department of Oncology, Centre for Cancer Genetic Epidemiology, University of Cambridge, Cambridge, UK; 470 Department of Public Health and Primary Care, Centre for Cancer Genetic Epidemiology, University of Cambridge, Cambridge, UK; 471 Prostate Cancer Canada, Toronto, ON Canada; 472 University of Cambridge, Cambridge, UK; 473 Department of Laboratory Medicine, Translational Cancer Research, Lund University Cancer Center at Medicon Village, Lund University, Lund, Sweden; 474 Heidelberg University, Heidelberg, Germany; 475 New BIH Digital Health Center, Berlin Institute of Health (BIH) and Charité - Universitätsmedizin Berlin, Berlin, Germany; 476 CIBER Epidemiología y Salud Pública (CIBERESP), Madrid, Spain; 477 Research Group on Statistics, Econometrics and Health (GRECS), UdG, Barcelona, Spain; 478 Quantitative Genomics Laboratories (qGenomics), Barcelona, Spain; 479 Icelandic Cancer Registry, Icelandic Cancer Society, Reykjavik, Iceland; 480 State Key Laboratory of Cancer Biology, and Xijing Hospital of Digestive Diseases, Fourth Military Medical University, Shaanxi, China; 481 Department of Medicine (DIMED), Surgical Pathology Unit, University of Padua, Padua, Italy; 482 Rigshospitalet, Copenhagen, Denmark; 483 Center for Cancer Genomics, National Cancer Institute, National Institutes of Health, Bethesda, MD USA; 484 Department of Biochemistry and Molecular Medicine, University of Montreal, Montreal, QC Canada; 485 Australian Institute of Tropical Health and Medicine, James Cook University, Douglas, QLD Australia; 486 Department of Neuro-Oncology, Istituto Neurologico Besta, Milano, Italy; 487 Bioplatforms Australia, North Ryde, NSW Australia; 488 Department of Pathology (Research), University College London Cancer Institute, London, UK; 489 Department of Surgical Oncology, Princess Margaret Cancer Centre, Toronto, ON Canada; 490 Department of Medical Oncology, Josephine Nefkens Institute and Cancer Genomics Centre, Erasmus Medical Center, Rotterdam, CN The Netherlands; 491 The University of Queensland Thoracic Research Centre, The Prince Charles Hospital, Brisbane, QLD Australia; 492 CIBIO/InBIO - Research Center in Biodiversity and Genetic Resources, Universidade do Porto, Vairão, Portugal; 493 HCA Laboratories, London, UK; 494 University of Liverpool, Liverpool, UK; 495 The Azrieli Faculty of Medicine, Bar-Ilan University, Safed, Israel; 496 Department of Neurosurgery, University of Florida, Gainesville, FL USA; 497 Department of Pathology, Graduate School of Medicine, University of Tokyo, Tokyo, Japan; 498 University of Milano Bicocca, Monza, Italy; 499 BGI-Shenzhen, Shenzhen, China; 500 Department of Pathology, Oslo University Hospital Ulleval, Oslo, Norway; 501 Center for Biomedical Informatics, Harvard Medical School, Boston, MA USA; 502 Department Biochemistry and Molecular Biomedicine, University of Barcelona, Barcelona, Spain; 503 Office of Cancer Genomics, National Cancer Institute, National Institutes of Health, Bethesda, MD USA; 504 Cancer Epigenomics, German Cancer Research Center (DKFZ), Heidelberg, Germany; 505 Department of Cancer Biology, The University of Texas MD Anderson Cancer Center, Houston, TX USA; 506 Department of Surgical Oncology, The University of Texas MD Anderson Cancer Center, Houston, TX USA; 507 Department of Computer Science, Yale University, New Haven, CT USA; 508 Department of Molecular Biophysics and Biochemistry, Yale University, New Haven, CT USA; 509 Program in Computational Biology and Bioinformatics, Yale University, New Haven, CT USA; 510 Center for Cancer Research, Massachusetts General Hospital, Boston, MA USA; 511 Department of Pathology, Massachusetts General Hospital, Boston, MA USA; 512 Department of Pathology, Memorial Sloan Kettering Cancer Center, New York, NY USA; 513 Division of Gastroenterology and Hepatology, Mayo Clinic, Rochester, MN USA; 514 University of Sydney, Sydney, NSW Australia; 515 University of Oxford, Oxford, UK; 516 Department of Surgery, Academic Urology Group, University of Cambridge, Cambridge, UK; 517 Department of Medicine II, University of Würzburg, Wuerzburg, Germany; 518 Sylvester Comprehensive Cancer Center, University of Miami, Miami, FL USA; 519 Institut Hospital del Mar d’Investigacions Mèdiques (IMIM), Barcelona, Spain; 520 Genome Integrity and Structural Biology Laboratory, National Institute of Environmental Health Sciences (NIEHS), Durham, NC USA; 521 St. Thomas’s Hospital, London, UK; 522 Osaka International Cancer Center, Osaka, Japan; 523 Department of Pathology, Skåne University Hospital, Lund University, Lund, Sweden; 524 Department of Medical Oncology, Beatson West of Scotland Cancer Centre, Glasgow, UK; 525 National Human Genome Research Institute, National Institutes of Health, Bethesda, MD USA; 526 Centre for Cancer Research, Victorian Comprehensive Cancer Centre, University of Melbourne, Melbourne, VIC Australia; 527 Department of Medicine, Section of Hematology/Oncology, University of Chicago, Chicago, IL USA; 528 German Center for Infection Research (DZIF), Partner Site Hamburg-Borstel-Lübeck-Riems, Hamburg, Germany; 529 Bioinformatics Research Centre (BiRC), Aarhus University, Aarhus, Denmark; 530 Department of Biotechnology, Ministry of Science and Technology, Government of India, New Delhi, Delhi India; 531 National Cancer Centre Singapore, Singapore, Singapore; 532 Brandeis University, Waltham, MA USA; 533 Department of Urologic Sciences, University of British Columbia, Vancouver, BC Canada; 534 Department of Internal Medicine, Stanford University, Stanford, CA USA; 535 The University of Texas Health Science Center at Houston, Houston, TX USA; 536 Imperial College NHS Trust, Imperial College, London, INY UK; 537 Senckenberg Institute of Pathology, University of Frankfurt Medical School, Frankfurt, Germany; 538 Department of Medicine, Division of Biomedical Informatics, UC San Diego School of Medicine, San Diego, CA USA; 539 Center for Precision Health, School of Biomedical Informatics, The University of Texas Health Science Center, Houston, TX USA; 540 Oxford Nanopore Technologies, New York, NY USA; 541 Institute of Medical Science, University of Tokyo, Tokyo, Japan; 542 Howard Hughes Medical Institute, University of California Santa Cruz, Santa Cruz, CA USA; 543 Wakayama Medical University, Wakayama, Japan; 544 Department of Internal Medicine, Division of Medical Oncology, Lineberger Comprehensive Cancer Center, University of North Carolina at Chapel Hill, Chapel Hill, NC USA; 545 University of Tennessee Health Science Center for Cancer Research, Memphis, TN USA; 546 Department of Histopathology, Salford Royal NHS Foundation Trust, Salford, UK; 547 Faculty of Biology, Medicine and Health, University of Manchester, Manchester, UK; 548 BIOPIC, ICG and College of Life Sciences, Peking University, Beijing, China; 549 Peking-Tsinghua Center for Life Sciences, Peking University, Beijing, China; 550 Children’s Hospital of Philadelphia, Philadelphia, PA USA; 551 Department of Bioinformatics and Computational Biology and Department of Systems Biology, The University of Texas MD Anderson Cancer Center, Houston, TX USA; 552 Karolinska Institute, Stockholm, Sweden; 553 The Donnelly Centre, University of Toronto, Toronto, ON Canada; 554 Department of Medical Genetics, College of Medicine, Hallym University, Chuncheon, South Korea; 555 Department of Experimental and Health Sciences, Institute of Evolutionary Biology (UPF-CSIC), Universitat Pompeu Fabra, Barcelona, Spain; 556 Health Data Science Unit, University Clinics, Heidelberg, Germany; 557 Massachusetts General Hospital Center for Cancer Research, Charlestown, MA USA; 558 Hokkaido University, Sapporo, Japan; 559 Department of Pathology and Clinical Laboratory, National Cancer Center Hospital, Tokyo, Japan; 560 Department of Genetics, University of North Carolina at Chapel Hill, Chapel Hill, NC USA; 561 Computational Biology, Leibniz Institute on Aging - Fritz Lipmann Institute (FLI), Jena, Germany; 562 University of Melbourne Centre for Cancer Research, Melbourne, VIC Australia; 563 University of Nebraska Medical Center, Omaha, NE USA; 564 Syntekabio Inc, Daejeon, South Korea; 565 Department of Pathology, Academic Medical Center, Amsterdam, AZ The Netherlands; 566 China National GeneBank-Shenzhen, Shenzhen, China; 567 Division of Molecular Genetics, German Cancer Research Center (DKFZ), Heidelberg, Germany; 568 Division of Life Science and Applied Genomics Center, Hong Kong University of Science and Technology, Clear Water Bay, Hong Kong, China; 569 Icahn School of Medicine at Mount Sinai, New York, NY USA; 570 Geneplus-Shenzhen, Shenzhen, China; 571 School of Computer Science and Technology, Xi’an Jiaotong University, Xi’an, China; 572 AbbVie, North Chicago, IL USA; 573 Institute of Pathology, Charité – University Medicine Berlin, Berlin, Germany; 574 Centre for Translational and Applied Genomics, British Columbia Cancer Agency, Vancouver, BC Canada; 575 Edinburgh Royal Infirmary, Edinburgh, UK; 576 Berlin Institute for Medical Systems Biology, Max Delbrück Center for Molecular Medicine, Berlin, Germany; 577 Department of Pediatric Immunology, Hematology and Oncology, University Hospital, Heidelberg, Germany; 578 German Cancer Research Center (DKFZ), Heidelberg, Germany; 579 Heidelberg Institute for Stem Cell Technology and Experimental Medicine (HI-STEM), Heidelberg, Germany; 580 Institute for Computational Biomedicine, Weill Cornell Medical College, New York, NY USA; 581 New York Genome Center, New York, NY USA; 582 Department of Urology, James Buchanan Brady Urological Institute, Johns Hopkins University School of Medicine, Baltimore, MD USA; 583 Department of Preventive Medicine, Graduate School of Medicine, The University of Tokyo, Tokyo, Japan; 584 Department of Molecular and Cellular Biology, Baylor College of Medicine, Houston, TX USA; 585 Department of Pathology and Immunology, Baylor College of Medicine, Houston, TX USA; 586 Michael E. DeBakey Veterans Affairs Medical Center, Houston, TX USA; 587 Technical University of Denmark, Lyngby, Denmark; 588 Department of Pathology, College of Medicine, Hanyang University, Seoul, South Korea; 589 Academic Unit of Surgery, School of Medicine, College of Medical, Veterinary and Life Sciences, University of Glasgow, Glasgow Royal Infirmary, Glasgow, UK; 590 Department of Pathology, Asan Medical Center, College of Medicine, Ulsan University, Songpa-gu, Seoul South Korea; 591 Science Writer, Garrett Park, MD USA; 592 International Cancer Genome Consortium (ICGC)/ICGC Accelerating Research in Genomic Oncology (ARGO) Secretariat, Ontario Institute for Cancer Research, Toronto, ON Canada; 593 University of Ljubljana, Ljubljana, Slovenia; 594 Department of Public Health Sciences, University of Chicago, Chicago, IL USA; 595 Research Institute, NorthShore University HealthSystem, Evanston, IL USA; 596 Department for Biomedical Research, University of Bern, Bern, Switzerland; 597 Centre of Genomics and Policy, McGill University and Génome Québec Innovation Centre, Montreal, QC Canada; 598 Carolina Center for Genome Sciences, University of North Carolina at Chapel Hill, Chapel Hill, NC USA; 599 Hopp Children’s Cancer Center (KiTZ), Heidelberg, Germany; 600 Pediatric Glioma Research Group, German Cancer Research Center (DKFZ), Heidelberg, Germany; 601 Cancer Research UK, London, UK; 602 Indivumed GmbH, Hamburg, Germany; 603 Genome Integration Data Center, Syntekabio, Inc, Daejeon, South Korea; 604 University Hospital Zurich, Zurich, Switzerland; 605 Clinical Bioinformatics, Swiss Institute of Bioinformatics, Geneva, Switzerland; 606 Institute for Pathology and Molecular Pathology, University Hospital Zurich, Zurich, Switzerland; 607 Institute of Molecular Life Sciences, University of Zurich, Zurich, Switzerland; 608 MRC Human Genetics Unit, MRC IGMM, University of Edinburgh, Edinburgh, UK; 609 Women’s Cancer Program at the Samuel Oschin Comprehensive Cancer Institute, Cedars-Sinai Medical Center, Los Angeles, CA USA; 610 Department of Biology, Bioinformatics Group, Division of Molecular Biology, Faculty of Science, University of Zagreb, Zagreb, Croatia; 611 Department for Internal Medicine II, University Hospital Schleswig-Holstein, Kiel, Germany; 612 Genetics and Molecular Pathology, SA Pathology, Adelaide, SA Australia; 613 Department of Gastric Surgery, National Cancer Center Hospital, Tokyo, Japan; 614 Department of Bioinformatics, Division of Cancer Genomics, National Cancer Center Research Institute, Tokyo, Japan; 615 A.A. Kharkevich Institute of Information Transmission Problems, Moscow, Russia; 616 Oncology and Immunology, Dmitry Rogachev National Research Center of Pediatric Hematology, Moscow, Russia; 617 Skolkovo Institute of Science and Technology, Moscow, Russia; 618 Department of Surgery, The George Washington University, School of Medicine and Health Science, Washington, DC USA; 619 Endocrine Oncology Branch, Center for Cancer Research, National Cancer Institute, National Institutes of Health, Bethesda, MD USA; 620 Melanoma Institute Australia, Macquarie University, Sydney, NSW Australia; 621 MIT Computer Science and Artificial Intelligence Laboratory, Massachusetts Institute of Technology, Cambridge, MA USA; 622 Tissue Pathology and Diagnostic Oncology, Royal Prince Alfred Hospital, Sydney, NSW Australia; 623 Cholangiocarcinoma Screening and Care Program and Liver Fluke and Cholangiocarcinoma Research Centre, Faculty of Medicine, Khon Kaen University, Khon Kaen, Thailand; 624 Controlled Department and Institution, New York, NY USA; 625 Englander Institute for Precision Medicine, Weill Cornell Medicine, New York, NY USA; 626 National Cancer Center, Gyeonggi, South Korea; 627 Department of Biochemistry, College of Medicine, Ewha Womans University, Seoul, South Korea; 628 Health Sciences Department of Biomedical Informatics, University of California San Diego, La Jolla, CA USA; 629 Research Core Center, National Cancer Centre Korea, Goyang-si, South Korea; 630 Department of Health Sciences and Technology, Sungkyunkwan University School of Medicine, Seoul, South Korea; 631 Samsung Genome Institute, Seoul, South Korea; 632 Breast Oncology Program, Dana-Farber/Brigham and Women’s Cancer Center, Boston, MA USA; 633 Department of Surgery, Memorial Sloan Kettering Cancer Center, New York, NY USA; 634 Division of Breast Surgery, Brigham and Women’s Hospital, Boston, MA USA; 635 Integrative Bioinformatics Support Group, National Institute of Environmental Health Sciences (NIEHS), Durham, NC USA; 636 Department of Clinical Science, University of Bergen, Bergen, Norway; 637 Center For Medical Innovation, Seoul National University Hospital, Seoul, South Korea; 638 Department of Internal Medicine, Seoul National University Hospital, Seoul, South Korea; 639 Institute of Computer Science, Polish Academy of Sciences, Warsawa, Poland; 640 Functional and Structural Genomics, German Cancer Research Center (DKFZ), Heidelberg, Germany; 641 Laboratory of Translational Genomics, Division of Cancer Epidemiology and Genetics, National Cancer Institute, , National Institutes of Health, Bethesda, MD USA; 642 Institute for Medical Informatics Statistics and Epidemiology, University of Leipzig, Leipzig, Germany; 643 Morgan Welch Inflammatory Breast Cancer Research Program and Clinic, The University of Texas MD Anderson Cancer Center, Houston, TX USA; 644 Department of Hematology and Oncology, Georg-Augusts-University of Göttingen, Göttingen, Germany; 645 Institute of Cell Biology (Cancer Research), University of Duisburg-Essen, Essen, Germany; 646 King’s College London and Guy’s and St. Thomas’ NHS Foundation Trust, London, UK; 647 Center for Epigenetics, Van Andel Research Institute, Grand Rapids, MI USA; 648 The University of Queensland Centre for Clinical Research, Royal Brisbane and Women’s Hospital, Herston, QLD Australia; 649 Department of Pediatric Oncology and Hematology, University of Cologne, Cologne, Germany; 650 University of Düsseldorf, Düsseldorf, Germany; 651 Department of Pathology, Institut Jules Bordet, Brussels, Belgium; 652 Institute of Biomedicine, Sahlgrenska Academy at University of Gothenburg, Gothenburg, Sweden; 653 Children’s Medical Research Institute, Sydney, NSW Australia; 654 ILSbio, LLC Biobank, Chestertown, MD USA; 655 Division of Genetics and Genomics, Boston Children’s Hospital, Harvard Medical School, Boston, MA USA; 656 Institute for Bioengineering and Biopharmaceutical Research (IBBR), Hanyang University, Seoul, South Korea; 657 Department of Statistics, University of California Santa Cruz, Santa Cruz, CA USA; 658 National Genotyping Center, Institute of Biomedical Sciences, Academia Sinica, Taipei, Taiwan; 659 Department of Vertebrate Genomics/Otto Warburg Laboratory Gene Regulation and Systems Biology of Cancer, Max Planck Institute for Molecular Genetics, Berlin, Germany; 660 McGill University and Genome Quebec Innovation Centre, Montreal, QC Canada; 661 biobyte solutions GmbH, Heidelberg, Germany; 662 Gynecologic Oncology, NYU Laura and Isaac Perlmutter Cancer Center, New York University, New York, NY USA; 663 Division of Oncology, Stem Cell Biology Section, Washington University School of Medicine, St. Louis, MO USA; 664 Department of Systems Biology, The University of Texas MD Anderson Cancer Center, Houston, TX USA; 665 Harvard University, Cambridge, MA USA; 666 Urologic Oncology Branch, Center for Cancer Research, National Cancer Institute, National Institutes of Health, Bethesda, MD USA; 667 University of Oslo, Oslo, Norway; 668 University of Toronto, Toronto, ON Canada; 669 Peking University, Beijing, China; 670 School of Life Sciences, Peking University, Beijing, China; 671 Leidos Biomedical Research, Inc, McLean, VA USA; 672 Hematology, Hospital Clinic, Institut d’Investigacions Biomèdiques August Pi i Sunyer (IDIBAPS), University of Barcelona, Barcelona, Spain; 673 Second Military Medical University, Shanghai, China; 674 Chinese Cancer Genome Consortium, Shenzhen, China; 675 Department of Medical Oncology, Beijing Hospital, Beijing, China; 676 Laboratory of Molecular Oncology, Key Laboratory of Carcinogenesis and Translational Research (Ministry of Education), Peking University Cancer Hospital and Institute, Beijing, China; 677 School of Medicine/School of Mathematics and Statistics, University of St. Andrews, St, Andrews, Fife UK; 678 Institute for Systems Biology, Seattle, WA USA; 679 Department of Biochemistry and Molecular Biology, Faculty of Medicine, University Institute of Oncology-IUOPA, Oviedo, Spain; 680 Institut Bergonié, Bordeaux, France; 681 Cancer Unit, MRC University of Cambridge, Cambridge, UK; 682 Department of Pathology and Laboratory Medicine, Center for Personalized Medicine, Children’s Hospital Los Angeles, Los Angeles, CA USA; 683 John Curtin School of Medical Research, Canberra, ACT Australia; 684 MVZ Department of Oncology, PraxisClinic am Johannisplatz, Leipzig, Germany; 685 Department of Information Technology, Ghent University, Ghent, Belgium; 686 Department of Plant Biotechnology and Bioinformatics, Ghent University, Ghent, Belgium; 687 Institute for Genomic Medicine, Nationwide Children’s Hospital, Columbus, OH USA; 688 Computational Biology Program, School of Medicine, Oregon Health and Science University, Portland, OR USA; 689 Department of Surgery, Duke University, Durham, NC USA; 690 Institució Catalana de Recerca i Estudis Avançats (ICREA), Barcelona, Spain; 691 Institut Català de Paleontologia Miquel Crusafont, Universitat Autònoma de Barcelona, Barcelona, Spain; 692 University of Glasgow, Glasgow, UK; 693 Institut d’Investigacions Biomèdiques August Pi i Sunyer (IDIBAPS), Barcelona, Spain; 694 Division of Oncology, Washington University School of Medicine, St. Louis, MO USA; 695 Department of Surgery and Cancer, Imperial College, London, INY UK; 696 Applications Department, Oxford Nanopore Technologies, Oxford, UK; 697 Department of Obstetrics, Gynecology and Reproductive Services, University of California San Francisco, San Francisco, CA USA; 698 Department of Biochemistry and Molecular Medicine, University California at Davis, Sacramento, CA USA; 699 STTARR Innovation Facility, Princess Margaret Cancer Centre, Toronto, ON Canada; 700 Discipline of Surgery, Western Sydney University, Penrith, NSW Australia; 701 Yale School of Medicine, Yale University, New Haven, CT USA; 702 Department of Genetics, Lineberger Comprehensive Cancer Center, University of North Carolina at Chapel Hill, Chapel Hill, NC USA; 703 Departments of Neurology and Neurosurgery, Henry Ford Hospital, Detroit, MI USA; 704 Precision Oncology, OHSU Knight Cancer Institute, Oregon Health and Science University, Portland, OR USA; 705 Institute of Pathology, University Medical Center Hamburg-Eppendorf, Hamburg, Germany; 706 Department of Health Sciences, Faculty of Medical Sciences, Kyushu University, Fukuoka, Japan; 707 Heidelberg Academy of Sciences and Humanities, Heidelberg, Germany; 708 Department of Clinical Pathology, University of Melbourne, Melbourne, VIC, Australia; 709 Department of Pathology, Roswell Park Cancer Institute, Buffalo, NY USA; 710 Department of Computer Science, University of Helsinki, Helsinki, Finland; 711 Institute of Biotechnology, University of Helsinki, Helsinki, Finland; 712 Organismal and Evolutionary Biology Research Programme, University of Helsinki, Helsinki, Finland; 713 Department of Obstetrics and Gynecology, Division of Gynecologic Oncology, Washington University School of Medicine, St. Louis, MO USA; 714 Penrose St. Francis Health Services, Colorado Springs, CO USA; 715 Institute of Pathology, Ulm University and University Hospital of Ulm, Ulm, Germany; 716 National Cancer Center, Tokyo, Japan; 717 Genome Institute of Singapore, Singapore, Singapore; 718 32Program in Computational Biology and Bioinformatics, Yale University, New Haven, CT USA; 719 German Cancer Aid, Bonn, Germany; 720 Programme in Cancer and Stem Cell Biology, Centre for Computational Biology, Duke-NUS Medical School, Singapore, Singapore; 721 The Chinese University of Hong Kong, Shatin, NT, Hong Kong China; 722 Fourth Military Medical University, Shaanxi, China; 723 The University of Cambridge School of Clinical Medicine, Cambridge, UK; 724 St. Jude Children’s Research Hospital, Memphis, TN USA; 725 University Health Network, Princess Margaret Cancer Centre, Toronto, ON Canada; 726 Center for Biomolecular Science and Engineering, University of California Santa Cruz, Santa Cruz, CA USA; 727 Department of Medicine, University of Chicago, Chicago, IL USA; 728 Department of Neurology, Mayo Clinic, Rochester, MN USA; 729 Cambridge Oesophagogastric Centre, Cambridge University Hospitals NHS Foundation Trust, Cambridge, UK; 730 Department of Computer Science, Carleton College, Northfield, MN USA; 731 Institute of Cancer Sciences, College of Medical Veterinary and Life Sciences, University of Glasgow, Glasgow, UK; 732 Department of Epidemiology, University of Alabama at Birmingham, Birmingham, AL USA; 733 HudsonAlpha Institute for Biotechnology, Huntsville, AL USA; 734 O’Neal Comprehensive Cancer Center, University of Alabama at Birmingham, Birmingham, AL USA; 735 Department of Pathology, Keio University School of Medicine, Tokyo, Japan; 736 Department of Hepatobiliary and Pancreatic Oncology, National Cancer Center Hospital, Tokyo, Japan; 737 Sage Bionetworks, Seattle, WA USA; 738 Lymphoma Genomic Translational Research Laboratory, National Cancer Centre, Singapore, Singapore; 739 Department of Clinical Pathology, Robert-Bosch-Hospital, Stuttgart, Germany; 740 Department of Cell and Systems Biology, University of Toronto, Toronto, ON Canada; 741 Department of Biosciences and Nutrition, Karolinska Institutet, Stockholm, Sweden; 742 Center for Liver Cancer, Research Institute and Hospital, National Cancer Center, Gyeonggi, South Korea; 743 Division of Hematology-Oncology, Samsung Medical Center, Sungkyunkwan University School of Medicine, Seoul, South Korea; 744 Samsung Advanced Institute for Health Sciences and Technology, Sungkyunkwan University School of Medicine, Seoul, South Korea; 745 Cheonan Industry-Academic Collaboration Foundation, Sangmyung University, Cheonan, South Korea; 746 NYU Langone Medical Center, New York, NY USA; 747 Department of Hematology and Medical Oncology, Cleveland Clinic, Cleveland, OH USA; 748 Department of Radiation Oncology, University of California San Francisco, San Francisco, CA USA; 749 Department of Health Sciences Research, Mayo Clinic, Rochester, MN USA; 750 Helen F. Graham Cancer Center at Christiana Care Health Systems, Newark, DE USA; 751 Heidelberg University Hospital, Heidelberg, Germany; 752 CSRA Incorporated, Fairfax, VA USA; 753 Research Department of Pathology, University College London Cancer Institute, London, UK; 754 Department of Research Oncology, Guy’s Hospital, King’s Health Partners AHSC, King’s College London School of Medicine, London, UK; 755 Faculty of Medicine and Health Sciences, Macquarie University, Sydney, NSW Australia; 756 University Hospital of Minjoz, INSERM UMR 1098, Besançon, France; 757 Spanish National Cancer Research Centre, Madrid, Spain; 758 Center of Digestive Diseases and Liver Transplantation, Fundeni Clinical Institute, Bucharest, Romania; 759 Cureline, Inc, South San Francisco, CA USA; 760 St. Luke’s Cancer Centre, Royal Surrey County Hospital NHS Foundation Trust, Guildford, UK; 761 Cambridge Breast Unit, Addenbrooke’s Hospital, Cambridge University Hospital NHS Foundation Trust and NIHR Cambridge Biomedical Research Centre, Cambridge, UK; 762 East of Scotland Breast Service, Ninewells Hospital, Aberdeen, UK; 763 Department of Genetics, Microbiology and Statistics, University of Barcelona, IRSJD, IBUB, Barcelona, Spain; 764 Department of Obstetrics and Gynecology, Medical College of Wisconsin, Milwaukee, WI USA; 765 Hematology and Medical Oncology, Winship Cancer Institute of Emory University, Atlanta, GA USA; 766 Department of Computer Science, Princeton University, Princeton, NJ USA; 767 Vanderbilt Ingram Cancer Center, Vanderbilt University, Nashville, TN USA; 768 Ohio State University College of Medicine and Arthur G. James Comprehensive Cancer Center, Columbus, OH USA; 769 Department of Surgery, Yokohama City University Graduate School of Medicine, Kanagawa, Japan; 770 Division of Chromatin Networks, German Cancer Research Center (DKFZ) and BioQuant, Heidelberg, Germany; 771 Research Computing Center, University of North Carolina at Chapel Hill, Chapel Hill, NC USA; 772 School of Molecular Biosciences and Center for Reproductive Biology, Washington State University, Pullman, WA USA; 773 Finsen Laboratory and Biotech Research and Innovation Centre (BRIC), University of Copenhagen, Copenhagen, Denmark; 774 Department of Laboratory Medicine and Pathobiology, University of Toronto, Toronto, ON Canada; 775 Department of Pathology, Human Oncology and Pathogenesis Program, Memorial Sloan Kettering Cancer Center, New York, NY USA; 776 University Hospital Giessen, Pediatric Hematology and Oncology, Giessen, Germany; 777 Oncologie Sénologie, ICM Institut Régional du Cancer, Montpellier, France; 778 Institute of Clinical Molecular Biology, Christian-Albrechts-University, Kiel, Germany; 779 Institute of Pathology, University of Wuerzburg, Wuerzburg, Germany; 780 Department of Urology, North Bristol NHS Trust, Bristol, UK; 781 SingHealth, Duke-NUS Institute of Precision Medicine, National Heart Centre Singapore, Singapore, Singapore; 782 Department of Computer Science, University of Toronto, Toronto, ON Canada; 783 Bern Center for Precision Medicine, University Hospital of Bern, University of Bern, Bern, Switzerland; 784 Englander Institute for Precision Medicine, Weill Cornell Medicine and New York Presbyterian Hospital, New York, NY USA; 785 Meyer Cancer Center, Weill Cornell Medicine, New York, NY USA; 786 Pathology and Laboratory, Weill Cornell Medical College, New York, NY USA; 787 Vall d’Hebron Institute of Oncology: VHIO, Barcelona, Spain; 788 General and Hepatobiliary-Biliary Surgery, Pancreas Institute, University and Hospital Trust of Verona, Verona, Italy; 789 National Centre for Biological Sciences, Tata Institute of Fundamental Research, Bangalore, India; 790 Indiana University, Bloomington, IN USA; 791 Department of Pathology, GZA-ZNA Hospitals, Antwerp, Belgium; 792 Analytical Biological Services, Inc, Wilmington, DE USA; 793 Sydney Medical School, University of Sydney, Sydney, NSW Australia; 794 cBio Center, Dana-Farber Cancer Institute, Harvard Medical School, Boston, MA USA; 795 Department of Cell Biology, Harvard Medical School, Boston, MA USA; 796 Advanced Centre for Treatment Research and Education in Cancer, Tata Memorial Centre, Navi Mumbai, Maharashtra India; 797 School of Environmental and Life Sciences, Faculty of Science, The University of Newcastle, Ourimbah, NSW Australia; 798 Department of Dermatology, University Hospital of Essen, Essen, Germany; 799 Bioinformatics and Omics Data Analytics, German Cancer Research Center (DKFZ), Heidelberg, Germany; 800 Department of Urology, Charité Universitätsmedizin Berlin, Berlin, Germany; 801 Martini-Clinic, Prostate Cancer Center, University Medical Center Hamburg-Eppendorf, Hamburg, Germany; 802 Department of General Internal Medicine, University of Kiel, Kiel, Germany; 803 German Cancer Consortium (DKTK), Partner site Berlin, Berlin, Germany; 804 Cancer Research Institute, Beth Israel Deaconess Medical Center, Boston, MA USA; 805 University of Pittsburgh, Pittsburgh, PA USA; 806 Department of Ophthalmology and Ocular Genomics Institute, Massachusetts Eye and Ear, Harvard Medical School, Boston, MA USA; 807 Center for Psychiatric Genetics, NorthShore University HealthSystem, Evanston, IL USA; 808 Van Andel Research Institute, Grand Rapids, MI USA; 809 Laboratory of Molecular Medicine, Human Genome Center, Institute of Medical Science, University of Tokyo, Tokyo, Japan; 810 Japan Agency for Medical Research and Development, Tokyo, Japan; 811 Korea University, Seoul, South Korea; 812 Murtha Cancer Center, Walter Reed National Military Medical Center, Bethesda, MD USA; 813 Human Genetics, University of Kiel, Kiel, Germany; 814 Department of Oncologic Pathology, Dana-Farber Cancer Institute, Harvard Medical School, Boston, MA USA; 815 Oregon Health and Science University, Portland, OR USA; 816 Center for RNA Interference and Noncoding RNA, The University of Texas MD Anderson Cancer Center, Houston, TX USA; 817 Department of Experimental Therapeutics, The University of Texas MD Anderson Cancer Center, Houston, TX USA; 818 Department of Gynecologic Oncology and Reproductive Medicine, The University of Texas MD Anderson Cancer Center, Houston, TX USA; 819 University Hospitals Coventry and Warwickshire NHS Trust, Coventry, UK; 820 Department of Radiation Oncology, Radboud University Nijmegen Medical Centre, Nijmegen, GA The Netherlands; 821 Institute for Genomics and Systems Biology, University of Chicago, Chicago, IL USA; 822 Clinic for Hematology and Oncology, St.-Antonius-Hospital, Eschweiler, Germany; 823 Computational and Systems Biology Program, Memorial Sloan Kettering Cancer Center, New York, NY USA; 824 University of Iceland, Reykjavik, Iceland; 825 Division of Computational Genomics and Systems Genetics, German Cancer Research Center (DKFZ), Heidelberg, Germany; 826 Dundee Cancer Centre, Ninewells Hospital, Dundee, UK; 827 Department for Internal Medicine III, University of Ulm and University Hospital of Ulm, Ulm, Germany; 828 Institut Curie, INSERM Unit 830, Paris, France; 829 Department of Gastroenterology and Hepatology, Yokohama City University Graduate School of Medicine, Kanagawa, Japan; 830 Department of Laboratory Medicine, Radboud University Nijmegen Medical Centre, Nijmegen, GA The Netherlands; 831 Division of Cancer Genome Research, German Cancer Research Center (DKFZ), Heidelberg, Germany; 832 Department of General Surgery, Singapore General Hospital, Singapore, Singapore; 833 Cancer Science Institute of Singapore, National University of Singapore, Singapore, Singapore; 834 Department of Medical and Clinical Genetics, Genome-Scale Biology Research Program, University of Helsinki, Helsinki, Finland; 835 East Anglian Medical Genetics Service, Cambridge University Hospitals NHS Foundation Trust, Cambridge, UK; 836 Irving Institute for Cancer Dynamics, Columbia University, New York, NY USA; 837 Institute of Molecular and Cell Biology, Singapore, Singapore; 838 Laboratory of Cancer Epigenome, Division of Medical Science, National Cancer Centre Singapore, Singapore, Singapore; 839 Universite Lyon, INCa-Synergie, Centre Léon Bérard, Lyon, France; 840 Department of Urology, Mayo Clinic, Rochester, MN USA; 841 Royal National Orthopaedic Hospital - Stanmore, Stanmore, Middlesex UK; 842 Department of Biochemistry, Genetics and Immunology, University of Vigo, Vigo, Spain; 843 Giovanni Paolo II / I.R.C.C.S. Cancer Institute, Bari, BA Italy; 844 Neuroblastoma Genomics, German Cancer Research Center (DKFZ), Heidelberg, Germany; 845 Fondazione Policlinico Universitario Gemelli IRCCS, Rome, Italy, Rome, Italy; 846 University of Verona, Verona, Italy; 847 Centre National de Génotypage, CEA - Institute de Génomique, Evry, France; 848 CAPHRI Research School, Maastricht University, Maastricht, ER The Netherlands; 849 Department of Biopathology, Centre Léon Bérard, Lyon, France; 850 Université Claude Bernard Lyon 1, Villeurbanne, France; 851 Core Research for Evolutional Science and Technology (CREST), JST, Tokyo, Japan; 852 Department of Biological Sciences, Laboratory for Medical Science Mathematics, Graduate School of Science, University of Tokyo, Yokohama, Japan; 853 Department of Medical Science Mathematics, Medical Research Institute, Tokyo Medical and Dental University (TMDU), Tokyo, Japan; 854 Cancer Ageing and Somatic Mutation Programme, Wellcome Sanger Institute, Hinxton, UK; 855 University Hospitals Birmingham NHS Foundation Trust, Birmingham, UK; 856 Centre for Cancer Research and Cell Biology, Queen’s University, Belfast, UK; 857 Breast Medical Oncology, The University of Texas MD Anderson Cancer Center, Houston, TX USA; 858 Department of Surgery, Johns Hopkins University School of Medicine, Baltimore, MD USA; 859 Department of Oncology-Pathology, Science for Life Laboratory, Karolinska Institute, Stockholm, Sweden; 860 School of Cancer Sciences, Faculty of Medicine, University of Southampton, Southampton, UK; 861 Department of Gene Technology, Tallinn University of Technology, Tallinn, Estonia; 862 Genetics and Genome Biology Program, SickKids Research Institute, The Hospital for Sick Children, Toronto, ON Canada; 863 Departments of Neurosurgery and Hematology and Medical Oncology, Winship Cancer Institute and School of Medicine, Emory University, Atlanta, GA USA; 864 Department of Clinical and Molecular Medicine, Faculty of Medicine and Health Sciences, Norwegian University of Science and Technology, Trondheim, Norway; 865 Argmix Consulting, North Vancouver, BC Canada; 866 Department of Information Technology, Ghent University, Interuniversitair Micro-Electronica Centrum (IMEC), Ghent, Belgium; 867 Nuffield Department of Surgical Sciences, John Radcliffe Hospital, University of Oxford, Oxford, UK; 868 Institute of Mathematics and Computer Science, University of Latvia, Riga, LV Latvia; 869 Discipline of Pathology, Sydney Medical School, University of Sydney, Sydney, NSW Australia; 870 Department of Applied Mathematics and Theoretical Physics, Centre for Mathematical Sciences, University of Cambridge, Cambridge, UK; 871 Department of Epidemiology and Biostatistics, Memorial Sloan Kettering Cancer Center, New York, NY USA; 872 Department of Statistics, Columbia University, New York, NY USA; 873 Department of Immunology, Genetics and Pathology, Science for Life Laboratory, Uppsala University, Uppsala, Sweden; 874 School of Electronic and Information Engineering, Xi’an Jiaotong University, Xi’an, China; 875 Department of Histopathology, Cambridge University Hospitals NHS Foundation Trust, Cambridge, UK; 876 Oxford NIHR Biomedical Research Centre, University of Oxford, Oxford, UK; 877 Georgia Regents University Cancer Center, Augusta, GA USA; 878 Wythenshawe Hospital, Manchester, UK; 879 Department of Genetics, Washington University School of Medicine, St.Louis, MO USA; 880 Department of Biological Oceanography, Leibniz Institute of Baltic Sea Research, Rostock, Germany; 881 Wellcome Centre for Human Genetics, University of Oxford, Oxford, UK; 882 Department of Molecular and Human Genetics, Baylor College of Medicine, Houston, TX USA; 883 Thoracic Oncology Laboratory, Mayo Clinic, Rochester, MN USA; 884 Institute for Genomic Medicine, Nationwide Children’s Hospital, Columbus, OH USA; 885 Department of Obstetrics and Gynecology, Division of Gynecologic Oncology, Mayo Clinic, Rochester, MN USA; 886 International Institute for Molecular Oncology, Poznań, Poland; 887 Poznan University of Medical Sciences, Poznań, Poland; 888 Genomics and Proteomics Core Facility High Throughput Sequencing Unit, German Cancer Research Center (DKFZ), Heidelberg, Germany; 889 NCCS-VARI Translational Research Laboratory, National Cancer Centre Singapore, Singapore, Singapore; 890 Edison Family Center for Genome Sciences and Systems Biology, Washington University, St. Louis, MO USA; 891 MRC-University of Glasgow Centre for Virus Research, Glasgow, UK; 892 Department of Medical Informatics and Clinical Epidemiology, Division of Bioinformatics and Computational Biology, OHSU Knight Cancer Institute, Oregon Health and Science University, Portland, OR USA; 893 School of Electronic Information and Communications, Huazhong University of Science and Technology, Wuhan, China; 894 Department of Applied Mathematics and Statistics, Johns Hopkins University, Baltimore, MD USA; 895 Department of Cancer Genome Informatics, Graduate School of Medicine, Osaka University, Osaka, Japan; 896 Institute of Computer Science, Heidelberg University, Heidelberg, Germany; 897 School of Mathematics and Statistics, University of Sydney, Sydney, NSW Australia; 898 Ben May Department for Cancer Research, University of Chicago, Chicago, IL USA; 899 Department of Human Genetics, University of Chicago, Chicago, IL USA; 900 Tri-Institutional PhD Program in Computational Biology and Medicine, Weill Cornell Medicine, New York, NY USA; 901 The First Affiliated Hospital, Xi’an Jiaotong University, Xi’an, China; 902 Department of Medicine and Therapeutics, The Chinese University of Hong Kong, Shatin, NT, Hong Kong China; 903 Department of Biostatistics, The University of Texas MD Anderson Cancer Center, Houston, TX USA; 904 Duke-NUS Medical School, Singapore, Singapore; 905 Department of Surgery, Ruijin Hospital, Shanghai Jiaotong University School of Medicine, Shanghai, China; 906 School of Computing Science, University of Glasgow, Glasgow, UK; 907 Division of Orthopaedic Surgery, Oslo University Hospital, Oslo, Norway; 908 Eastern Clinical School, Monash University, Melbourne, VIC Australia; 909 Epworth HealthCare, Richmond, VIC Australia; 910 Department of Biostatistics and Computational Biology, Dana-Farber Cancer Institute and Harvard Medical School, Boston, MA USA; 911 Department of Biomedical Informatics, College of Medicine, The Ohio State University, Columbus, OH USA; 912 The Ohio State University Comprehensive Cancer Center (OSUCCC – James), Columbus, OH USA; 913 The University of Texas School of Biomedical Informatics (SBMI) at Houston, Houston, TX USA; 914 Department of Biostatistics, University of North Carolina at Chapel Hill, Chapel Hill, NC USA; 915 Department of Biochemistry and Molecular Genetics, Feinberg School of Medicine, Northwestern University, Chicago, IL USA; 916 Faculty of Medicine and Health, University of Sydney, Sydney, NSW Australia; 917 Department of Pathology, Erasmus Medical Center Rotterdam, Rotterdam, GD The Netherlands; 918 Division of Molecular Carcinogenesis, The Netherlands Cancer Institute, Amsterdam, CX The Netherlands; 919 Institute of Molecular Life Sciences and Swiss Institute of Bioinformatics, University of Zurich, Zurich, Switzerland

**Keywords:** Cancer genomics, Computational biology and bioinformatics, Cancer

## Abstract

Cancers require telomere maintenance mechanisms for unlimited replicative potential. They achieve this through TERT activation or alternative telomere lengthening associated with ATRX or DAXX loss. *Here, as part of the ICGC/TCGA Pan-Cancer Analysis of Whole Genomes (PCAWG) Consortium*, we dissect whole-genome sequencing data of over 2500 matched tumor-control samples from 36 different tumor types aggregated within the ICGC/TCGA Pan-Cancer Analysis of Whole Genomes (PCAWG) Consortium to characterize the genomic footprints of these mechanisms. While the telomere content of tumors with *ATRX* or *DAXX* mutations (ATRX/DAXX^trunc^) is increased, tumors with *TERT* modifications show a moderate decrease of telomere content. One quarter of all tumor samples contain somatic integrations of telomeric sequences into non-telomeric DNA. This fraction is increased to 80% prevalence in ATRX/DAXX^trunc^ tumors, which carry an aberrant telomere variant repeat (TVR) distribution as another genomic marker. The latter feature includes enrichment or depletion of the previously undescribed singleton TVRs TTCGGG and TTTGGG, respectively. Our systematic analysis provides new insight into the recurrent genomic alterations associated with telomere maintenance mechanisms in cancer.

## Abstract (web summary)

In somatic cells the mechanisms maintaining the chromosome ends are normally inactivated; however, cancer cells can re-activate these pathways to support continuous growth. Here, the authors characterize the telomeric landscapes across tumour types and identify genomic alterations associated with different telomere maintenance mechanisms.

## Introduction

Telomeres are nucleoprotein complexes at the ends of chromosomes that prevent DNA degradation and genome instability^1^. The typically 10–15 kb long chromosome termini are composed of long stretches of TTAGGG (t-type) repeat arrays with an increasing number of variants toward proximal, subtelomeric regions, the most common being TGAGGG (g-type), TCAGGG (c-type), and TTGGGG (j-type) repeats^2,3^.

Telomeres play an important role in cellular aging, as they are shortened with each cell division and finally trigger a DNA damage response resulting in senescence^4,5^. To avoid this permanent growth arrest, cells with unlimited proliferative potential need to extend their telomeres. In humans, telomeric DNA is synthesized by telomerase, an enzyme that is composed of the reverse transcriptase TERT and the RNA template TERC. This complex is active in the germline and stem cells, but absent in most somatic cells^6^. Telomerase is upregulated in ~85% of human cancers by different genetic aberrations, including *TERT* amplifications^7^, rearrangements^8^, or mutations in the *TERT* promoter^9,10^. The remaining tumors employ an alternative lengthening of telomeres (ALT) pathway, which is based on DNA recombination of telomeric sequences^11^. Details on the ALT mechanism remain elusive, but it has been associated with loss-of-function mutations in the chromatin remodeling genes *ATRX* (α-thalassaemia/mental retardation syndrome X-linked) and *DAXX* (death domain-associated protein)^12^. Telomeres of ALT cells characteristically have heterogeneous lengths and contain a range of telomere variant repeats (TVRs)^13–15^. Other hallmarks of ALT include ALT-associated promyelocytic leukemia nuclear bodies, abundance of extrachromosomal telomeric repeats of various forms (such as C-circles), and genome instability^11,16^.

While normally located at the chromosome termini, telomere sequences are also found within chromosomes. As such, interstitial telomeric sequences with large blocks of telomere repeats exist in humans and other species, which probably arose from ancestral genome rearrangements or other evolutionary events^17^. Recently, also ALT-specific, targeted telomere insertions into chromosomes have been described that lead to genomic instability^18^. Another source for unexpected telomere repeat occurrence is the stabilizing function of telomeres at broken chromosomes. After a double-strand break, telomeres can be added de novo to the unprotected break sites (“telomere healing”)^19,20^ or acquired from other chromosomal positions (“telomere capture”)^21,22^.

The here presented study was conducted within the scope of the ICGC/TCGA Pan-Cancer Analysis of Whole Genomes (PCAWG) Consortium, which aggregated whole-genome sequencing (WGS) data from 2658 cancers across 38 tumor types generated by the ICGC and TCGA projects. This data was reanalyzed with standardized, high-accuracy pipelines to align to the human genome (reference build hs37d5), and identify germline variants and somatically acquired mutations, as described in ref. ^23^.

Here, we characterize the telomere landscape of 2519 tumor samples from 36 different tumor types using the WGS alignments, somatic mutation, and chromothripsis calls provided by the PCAWG Consortium^23,24^. Besides determining telomere content and searching for mutations associated with different telomere maintenance mechanisms (TMMs), we systematically detect 2683 somatic telomere insertions and show that different TMMs are associated with the enrichment of previously undescribed singleton TVRs.

## Results

### Telomere content across cohorts

Due to the repetitive nature of telomere sequences, short sequencing reads from telomeres cannot be uniquely aligned to individual chromosomes. However, a mean telomere content for the tumor as a whole can be estimated from the number of reads containing telomere sequences^25^. Here, we extracted reads containing at least six telomere repeats per 100 bases, allowing the canonical telomere repeat TTAGGG and the three most common TVRs TCAGGG, TGAGGG, and TTGGGG. The telomere content was defined as the number of unaligned telomere reads normalized by sequencing coverage and GC content. Of the 2583 high-quality tumor samples available in PCAWG, we selected those from donors with a single tumor sample. From each donor, a control sample was available. In most cases this consisted of a blood sample, but could also stem from tumor-adjacent or other tissue^23^. The telomere content was determined for the remaining 2519 tumor samples and matched controls from 36 different tumor types. Several of these tumor types were not covered in a recent pan-cancer overview of telomere lengths^26^, including medulloblastoma, pilocytic astrocytoma, chronic lymphocytic leukemia, pancreatic endocrine cancers, benign bone cancer, and osteosarcoma. All relevant donor information and results used in this study are summarized in Supplementary Data 1.

Telomere content of the controls anticorrelated with age (*r* = −0.36, Spearman correlation; Supplementary Fig. 1a). However, this age effect only has a low contribution to the strong correlation between the telomere content of the tumor and control samples (*r* = 0.47 and *r*_partial_ = 0.46 given the patient age, Spearman correlation, Supplementary Fig. 1b). Thus, the association of tumor and control telomere content must mainly be caused by other genetic^27,28^, environmental^29^, or technical factors^26^. We normalized for these contributions by computing the ratio of tumor and control telomere content per individual.

**Fig. 1:**
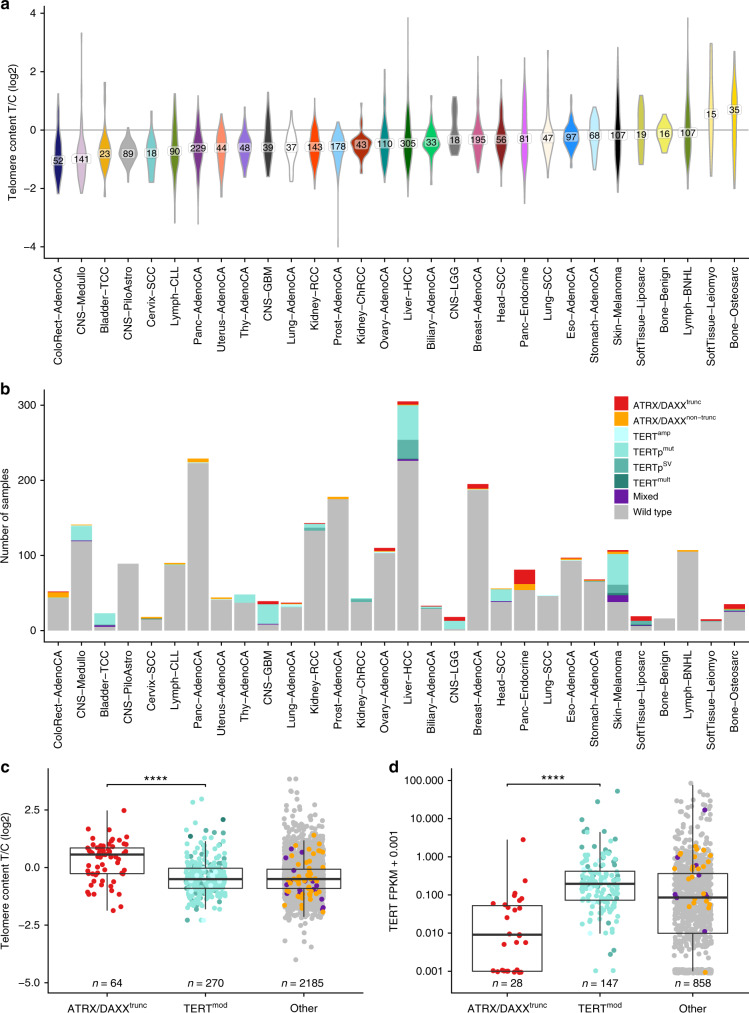
Telomere content is increased in ATRX/DAXX^trunc^ samples. **a** Overview of the telomere content distribution of all analyzed tumor types. The number of samples in each tumor type is indicated. Cohorts with sample sizes <15 are not shown. **b** TMM-associated mutations in different tumor types. **c** Telomere content in samples with different TMM-associated mutations. **d** *TERT* expression in samples with different TMM-associated mutations. The center lines of the boxplots are the medians, the bounds of the boxes represent the first and third quartiles, the upper and lower whiskers extend from the hinge to the largest or smallest value, respectively, no further than 1.5 × IQR from the hinge (where IQR is the interquartile range, or distance between the first and third quartiles). ****p < 0.0001, Wilcoxon rank-sum tests.

Most tumor samples had a lower telomere content than the matched control (Fig. 1a). However, there were systematic differences between the different tumor types. Among those with the highest telomere content increase were osteosarcomas and leiomyosarcomas (median telomere content tumor/control log2 ratios = 0.7 and 0.6, respectively). A particularly low telomere content was found in colorectal adenocarcinoma and medulloblastoma (median telomere content tumor/control log2 ratios = −1.0).

### Prevalence of TMM-associated mutations

Different types of mutations in *ATRX* or *DAXX*, and at the *TERT* locus have been associated with ALT and telomerase activation, respectively. We therefore searched for these types of somatic mutations to infer the active TMM in a given tumor. Somatic mutations in *ATRX*, *DAXX*, or *TERT* were found in 16% of tumor samples. In total, 64 tumor samples had truncating *ATRX* (*n* = 53) or *DAXX* alterations (*n* = 11), and are referred to as ATRX/DAXX^trunc^ in the following analysis. Of note, 10 of the 11 *DAXX* alterations were found in pancreatic endocrine tumors, while *ATRX* mutations were seen in a wider variety of entities. An additional 46 samples had nontruncating *ATRX/DAXX* simple nucleotide variants. *TERT* alterations (TERT^mod^) were detected in 270 of the 2519 tumor samples (11%). The latter group comprised 198 activating C228T or C250T promoter mutations (of which 132 were obtained from the PCAWG simple nucleotide variant consensus calls and the remaining were detected with a targeted approach), 11 amplifications leading to at least six additional *TERT* copies, 55 structural variations within 20 kb upstream of *TERT* (TERTp^SV^), and 6 samples with more than one of these modifications. Additionally, 18 tumor samples had both *ATRX/DAXX* truncating or other missense mutations and *TERT* alterations.

“Enhancer hijacking” near the *TERT* transcription start site (TSS) has been described in neuroblastoma^8^ and has recently been indicated in further cancer types^26^. In our data set, the strikingly focal distribution of structural variations upstream of the *TERT* TSS also points to this phenomenon (Supplementary Fig. 2). Further evidence is given by the direct overlap of 40% (*n* = 25/62) of the juxtaposed positions within 20 kb upstream of the *TERT* TSS with enhancers from the dbSUPER database^30^. In contrast, only 13% (*n* = 9/69) of the juxtaposed positions between 20 and 1000 kb corresponded to a predicted super-enhancer. Besides in melanoma (13% prevalence), chromophobe renal cell (9%), hepatocellular (9%), bladder transitional cell (4%), biliary (3%), and renal clear cell carcinoma (3%), previously undescribed TERTp^SV^ in osteosarcoma (9%) and stomach adenocarcinoma (1%) were found. Moreover, the subtype-specific histological classification available in this study showed that TERTp^SV^ were more frequent in liposarcoma (32%) than in leiomyosarcoma (7%).

The tumor types with the highest prevalence of ATRX/DAXX^trunc^ mutations were liposarcomas (32%), adult lower grade gliomas (28%), pancreatic endocrine tumors (23%), and osteosarcoma (17%; Fig. 1b and Supplementary Fig. 3a, b), all of which have previously been associated with ALT^12,31^. TERT^mod^ were most prevalent in transitional cell bladder cancer (70%), glioblastoma (67%), lower grade gliomas (61%), and melanoma (51%).

The telomere content in TERT^mod^ samples differed significantly from that in ATRX/DAXX^trunc^ samples (*p* = 1.1 × 10^−9^, Wilcoxon rank-sum test; Fig. 1c, a detailed overview is shown in Supplementary Fig. 4a). On average, telomere content was gained in ATRX/DAXX^trunc^ (mean telomere content tumor/control log2 ratio = 0.3), while telomere sequences were lost in TERT^mod^ samples (mean telomere content tumor/control log2 ratio = −0.4). Samples with nontruncating *ATRX/DAXX* simple nucleotide variants had a similar telomere content as TERT^mod^ samples (*p* > 0.05, Wilcoxon rank-sum test), suggesting that most of the nontruncating *ATRX/DAXX* mutations are passenger events. In TERT^mod^ samples and samples with unknown TMM, the telomere content correlated with *TERT* expression (*r* = 0.20, Pearson correlation; *p* = 4.1 × 10^−10^, significance of fitted linear regression model) and *TERT* expression was significantly higher in TERT^mod^ samples than in ATRX/DAXX^trunc^ samples (*p* = 1.3 × 10^−9^, Wilcoxon rank-sum test; Fig. 1d, detailed overviews are shown in Supplementary Figs. 3c and 4b).

### High amount of telomere insertions in ATRX/DAXX^trunc^ tumors

**Fig. 2:**
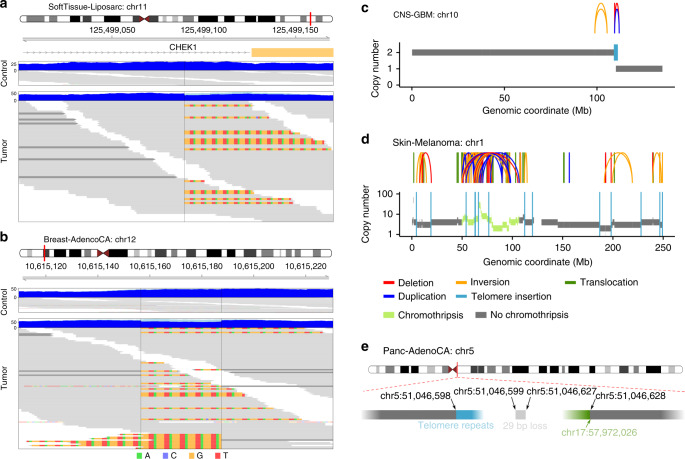
Examples of telomere insertions. **a** One-sided telomere insertion in liposarcoma sample SP121774. Blue tracks show the sequencing coverage; light blue represents clipped sequences. Individual reads are grey and clipped bases are colored. Dark grey reads represent the nontelomeric end of a discordant read pair. **b** Two-sided telomere insertion in breast adenocarcinoma sample SP5636. Nontelomeric clipped bases are transparent. **c** One-sided telomere insertion accompanied by copy number loss of the adjacent chromosome end in glioblastoma sample SP29559. Arches represent structural variations. **d** Multiple telomere insertions in a chromosome that underwent chromothripsis in melanoma sample SP124441. **e** One-sided telomere insertion accompanied by a translocation of the adjacent chromosome segment in pancreatic adenocarcinoma sample SP125764.

To find insertions of telomeres into nontelomeric regions of the genome, we searched for tumor-specific discordant paired-end reads, where one end maps to the chromosome and the other end is telomeric. Exact positions of the insertions were determined from reads spanning the junction site and visual inspection (Fig. 2).

**Fig. 3:**
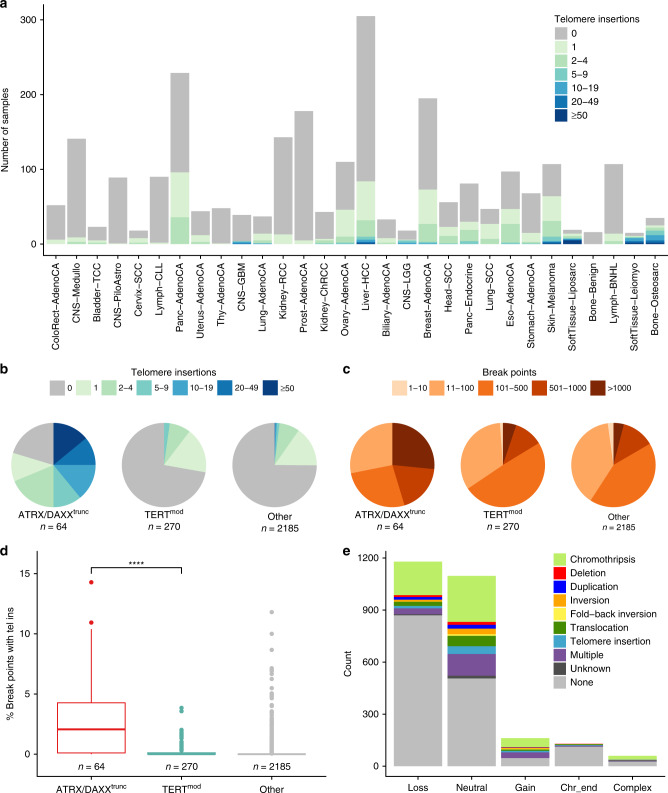
Insertion of telomere sequences into nontelomeric chromosomal regions. **a** Number of telomere insertions in samples of different tumor types. The tumor types are sorted by mean telomere content tumor/control log2 ratios. Cohorts with sample sizes <15 are not shown. **b** Number of telomere insertions in samples with different TMM-associated mutations. **c** Number of breakpoints in samples with different TMM-associated mutations. **d** Percent of breakpoints coinciding with telomere insertions in samples with different TMM-associated mutations. The center line of the boxplot is the median, the bounds of the box represent the first and third quartiles, the upper and lower whiskers extend from the hinge to the largest or smallest value, respectively, no further than 1.5 × IQR from the hinge (where IQR is the interquartile range, or distance between the first and third quartiles). ****p < 0.0001, Wilcoxon rank-sum test. **e** Copy number changes of adjacent segments accompanying telomere insertions. “Complex” means that the copy numbers between segments differ in more than four copies. Overlaps with regions of chromothripsis are indicated. For telomere insertions that did not overlap with regions of chromothripsis, structural variations, or additional telomere insertions within 10 kb are indicated.

Overall, 2683 telomere insertions were detected. These were distributed unevenly between samples and different tumor types (Fig. 3a). Telomere insertions were found in 27% of the tumor samples, with counts ranging between 1 and 228 telomere insertion events. The tumor types with the highest amount of telomere insertions per tumor sample were liposarcoma, leiomyosarcoma, and osteosarcoma, all of which also had a relatively high mean telomere content. In fact, the number of telomere insertions positively correlated with the telomere content (*r* = 0.19, Spearman correlation). Moreover, the number of telomere insertions was associated with the number of genomic breakpoints in the sample (*r* = 0.38, Spearman correlation). To test for a synergistic effect, linear models that predict telomere insertions from telomere content and breakpoint abundance with and without an interaction term were computed. The models with the interaction term (*p* = 8.8 × 10^−234^) performed substantially better than purely additive models (*p* = 5.8 × 10^−90^).

There was clearly a higher percentage of samples with telomere insertions in ATRX/DAXX^trunc^ tumors (80%) than TERT^mod^ tumors (28%; Fig. 3b). As expected, ATRX/DAXX^trunc^ samples also had a higher number of breakpoints (mean = 733) than TERT^mod^ samples (mean = 291; Fig. 3c). Overall, the fraction of genomic breakpoints overlapping with telomere insertion sites was significantly higher in ATRX/DAXX^trunc^ than TERT^mod^ samples (*p* = 1.7 × 10^−20^, Wilcoxon rank-sum test; Fig. 3d). In agreement with the high breakpoint frequency, chromothripsis (numerous chromosomal rearrangements occurring in a single event)^32^ was more prevalent in the ATRX/DAXX^trunc^ samples (59%) compared to TERT^mod^ samples (34%) and samples without ATRX/DAXX^trunc^ and TERT^mod^ mutations (29%). Similarly, ATRX/DAXX^trunc^ samples were more likely to have an autosomal breakage-fusion-bridge (BFB) event (44%) than the remaining samples (TERT^mod^: 31%, other: 32%). In ATRX/DAXX^trunc^ samples, autosomal chromosome arms that showed evidence for BFB cycles and chromothripsis had the highest incidence of telomere insertions (Supplementary Fig. 5).

Correlation analysis of telomere insertions and mutations in telomere maintenance-associated genes from the TelNet database^33^ [http://www.cancertelsys.org/telnet] revealed significant association with *TP53* (*q* = 1.9 × 10^−42^), *ATRX* (*q* = 2.6 × 10^−6^), *PLCB2* (*q* = 7.8 × 10^−4^), *MEN1* (*q* = 0.017), *TSSC4* (*q* = 0.017), *RB1* (*q* = 0.018), *DAXX* (*q* = 0.019), and *ABCC8* mutations (*q* = 0.04, Wilcoxon rank-sum tests after Benjamini–Hochberg correction). Most of these genes have been implicated in the maintenance of telomere length or structure in humans (Supplementary Table 1). The exceptions are *PLCB2* and *ABCC8*, whose homologues have so far only been reported in association with telomere length regulation in yeast^34,35^.

The detected telomere insertions were scattered across different chromosomes and regions within the chromosome (Supplementary Fig. 6). No clear preferential insertion sites were identified, but several de novo telomere junctions occurred at the chromosome ends (5% within 50 kb of the first or last chromosomal segment). A total of 44% of the telomere insertions were in genes, and 8% of these disrupted exons. Several tumor suppressor genes were affected, e.g., *CHEK1* encoding for a protein involved in cell cycle arrest upon DNA damage^36^ (Fig. 2a).

Of note, patterns of microhomology were observed in 79% of telomere insertions with t-type repeats at the junction site (Supplementary Fig. 7).

### Frequent copy number losses at telomere insertion sites

Most of the telomere insertions were one-sided (98%), i.e., telomere sequences were only attached to one side of the breakpoint (Fig. 2a). Telomere insertions were defined as two-sided, if there was a second telomere insertion event downstream in the opposite orientation (Fig. 2b). Two-sided telomere insertions can occur via a telomere sequence that bridges two chromosome fragments or, alternatively, telomere sequences are independently fused to both ends of the chromosome break. Reads supporting the first scenario were found in 14 of the 25 two-sided telomere insertions pairings. For the other cases, the inserted repeat sequence was too long to distinguish between the two scenarios.

Because so many breakpoints were one-sided, we investigated the fate of the corresponding broken fragment using complementary information from copy number changes and structural variation annotation (Fig. 3e). As expected, one-sided telomere insertions coincided most frequently with copy number loss of the adjacent segment (46%, Fig. 2c). In contrast, copy number gains of the fragment were rare (6%). Surprisingly, telomere insertions were frequently located at copy-number neutral sites (42%). Overlaps with regions of chromothripsis were found for 25% and structural variations without chromothripsis overlap (including telomere insertions) were detected near the insertion site for 28% of the copy-number neutral cases (Fig. 2d, e). The remaining telomere insertions at copy-number neutral sites are likely to be subclonal (Supplementary Fig. 8a) or have undetected structural variations nearby (Supplementary Fig. 8b).

### Occasional TERRA expression at telomere insertions

ALT-positive tumors have been associated with elevated levels of long noncoding telomeric repeat-containing RNA (TERRA)^16^. We searched for TERRA expression in the RNA-sequencing data of 867 tumor samples. In line with the results of Barthel et al.^26^, TERRA levels were higher in ATRX/DAXX^trunc^ compared to TERT^mod^ samples (*p* = 5.0 × 10^−7^, Wilcoxon rank-sum test, Supplementary Fig. 9). In 16 samples, evidence for TERRA expression at telomere insertion sites was found. For most of these, the number of split reads supporting TERRA expression was low (between 1 and 8 reads). However, 146 TERRA reads expressed from only two telomere insertion sites were detected in an ATRX/DAXX^trunc^ liposarcoma sample, making up almost 6% of its total TERRA read count. This percentage is likely to be notably higher, as the short read length does not allow assignment of the total number of TERRA reads stemming from these telomere insertions. Thus, TERRA is not exclusively transcribed from TSSs in the subtelomeric region but can also arise from telomere insertions. Of note, these telomere insertion transcripts do not always contain the canonical UUAGGG repeat but can also be composed of the reverse complement CCCUAA.

### Enrichment of singleton TVRs in ATRX/DAXX^trunc^ samples

It has previously been shown that ALT leads to an increased integration of TVRs into telomeres, the most common ones being hexamers of the type NNNGGG^15^. To detect differences in the telomere composition of ATRX/DAXX^trunc^ and TERT^mod^ tumors, we therefore searched for NNNGGG repeats in telomere reads. The most frequent TVRs across all tumor samples were TGAGGG, TCAGGG, and TTGGGG (Supplementary Fig. 10), which are known to be enriched in proximal telomeric regions^2,3^.

These and the seven other most frequent TVRs (TAAGGG, GTAGGG, CATGGG, TTCGGG, CTAGGG, TTTGGG, and ATAGGG) were chosen to search for common telomere repeat combinations. For this, the neighboring 18 base pairs on either side of the TVRs were determined (Supplementary Data 2). Most TVRs were surrounded by many different pattern combinations (e.g., TTGGGG). Others were dominated by a certain repeat context, which was similar in ATRX/DAXX^trunc^ and TERT^mod^ tumors (e.g., CATGGG or ATAGGG). However, TTCGGG stood out, as 41% of the TVRs in ATRX/DAXX^trunc^ samples were surrounded by canonical t-type repeats, whereas this context was observed for only 4% of TTCGGG TVRs in TERT^mod^ tumors.

**Fig. 4:**
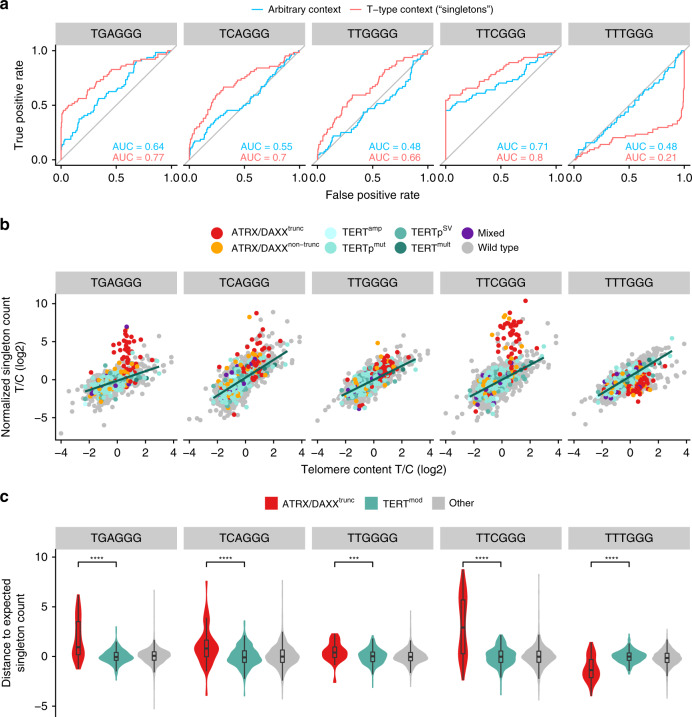
Singleton TVRs enriched or depleted in ATRX/DAXX^trunc^ samples. **a** Receiver operating characteristic for the classification of samples with ALT-associated mutations from telomere variant repeats. Red: no specific sequence context required. Blue: singletons ((TTAGGG)_3_-NNNGGG-(TTAGGG)_3_). The more the area under the curve (AUC) deviates from 0.5, the better the repeat occurrence distinguishes ATRX/DAXX^trunc^ from TERT^mod^ samples. **b** Pattern count tumor/control log2 ratios of all patients plotted against telomere content tumor/control log2 ratios for selected singletons. The regression line through the TERT^mod^ samples is shown in green and is defined as the expected pattern count in the following. **c** Distance to the expected singleton repeat count in ATRX/DAXX^trunc^ and TERT^mod^ samples. The center line of the boxplot is the median, the bounds of the box represent the first and third quartiles, the upper and lower whiskers extend from the hinge to the largest or smallest value, respectively, no further than 1.5 × IQR from the hinge (where IQR is the interquartile range, or distance between the first and third quartiles). ****p < 0.0001; ***p < 0.001, Wilcoxon rank-sum tests after Bonferroni correction. The profiles of all analyzed patterns are shown in Supplementary Figs. 11 and 13.

Following up on this observation, we compared variant hexamers surrounded by at least three t-type repeats on either side (“singletons”) to TVRs in an arbitrary sequence context. This revealed that singletons are generally well suited to distinguish ATRX/DAXX^trunc^ from TERT^mod^ samples (Fig. 4a, an overview of all patterns is shown in Supplementary Fig. 11). The remaining variant analysis therefore focused on such TVR singletons. CATGGG was excluded as it did not occur as singletons. For the other TVRs, the median of absolute counts varied between 12 and 100, but counts in individual tumor samples reached >10,000 (Supplementary Fig. 12).

As expected, normalized singleton repeat counts generally rose with increasing telomere content (Fig. 4b, an overview of all patterns is shown in Supplementary Fig. 13). However, TGAGGG, TCAGGG, TTGGGG, and TTCGGG singletons had significantly higher counts than expected in ATRX/DAXX^trunc^ compared to TERT^mod^ samples (*p* = 7.3 × 10^−11^, 7.9 × 10^−6^, 5.6 × 10^−4^, and 5.8 × 10^−13^, respectively, Wilcoxon rank-sum test after Bonferroni correction; Fig. 4c). Especially, TGAGGG and TTCGGG seemed to be highly interspersed in a subset of ATRX/DAXX^trunc^ tumors. In contrast, TTTGGG singletons were observed less frequently in ATRX/DAXX^trunc^ tumors (*p* = 3.8 × 10^−12^, Wilcoxon rank-sum test after Bonferroni correction).

This seemingly ALT-specific TVR enrichment or depletion occurred in different tumor types, with the highest prevalence in leiomyosarcomas (60%), pancreatic endocrine tumors (42%), liposarcomas (37%), osteosarcomas (29%), and lower grade gliomas (28%; Supplementary Table 2). In the ATRX/DAXX^trunc^ samples, singleton TVR occurrences correlated with each other (Supplementary Fig. 14). The strongest correlations were between TGAGGG occurrence and TTCGGG and TTGGGG singletons (*r* = 0.57 and *r* = 0.58, respectively, Spearman correlation).

### ALT prediction

ALT has several different hallmarks with which it can be reliably identified^11^. However, most of these are not detectable in short-read WGS data. Using ATRX/DAXX^trunc^ as indicators of ALT, we have shown several possible TMM classification features based on WGS. Most ATRX/DAXX^trunc^ samples are already separated well from TERT^mod^ samples by nonsupervised clustering of normalized TGAGGG, TCAGGG, TTGGGG, TTCGGG, and TTTGGG singleton repeat counts (Supplementary Fig. 15). As expected, the clusters of ATRX/DAXX^trunc^ samples had a high telomere content and a high number of telomere insertions relative to the total number of breakpoints. These features were further used to build a random forest classifier distinguishing ATRX/DAXX^trunc^ from TERT^mod^ samples (area under the curve: 0.95; sensitivity: 0.73; specificity: 0.99; all after 10-fold cross-validation). The variables with the highest importance for the classification were the divergence of observed TTTGGG and TTCGGG singleton TVRs from the expected count, the number of breakpoints and the number of telomere insertions (Supplementary Table 3). It may be pivotal for further understanding of this mechanism to determine the causal relationship between these features and the ALT phenotype.

The scores resulting from the classifier can be interpreted as an ALT probability. As expected, ATRX/DAXX^trunc^ had a high ALT probability (mean = 0.91), while TERT^mod^ samples had a low ALT probability (mean = 0.13, Supplementary Fig. 16). A total of 17 samples without ATRX/DAXX^trunc^ mutations had an ALT probability of over 0.9, of which three had nontruncating ATRX/DAXX mutations and one sample had a frameshift insertion in *ATRX* and a *TERT* amplification (11 TERT copies, triploid). Across the entire dataset, most samples had a low ALT probability (Supplementary Fig. 17), suggesting that their TMM is telomerase based. This included some samples with ATRX/DAXX missense mutations, suggesting that the mutations in those samples may be more of a passenger event than functionally relevant. Tumor types with a high ALT probability were leiomyosarcoma, osteosarcoma, pancreatic endocrine tumors, and liposarcomas, in keeping with the known high prevalence of ALT in these entities^37–39^.

## Discussion

**Fig. 5:**
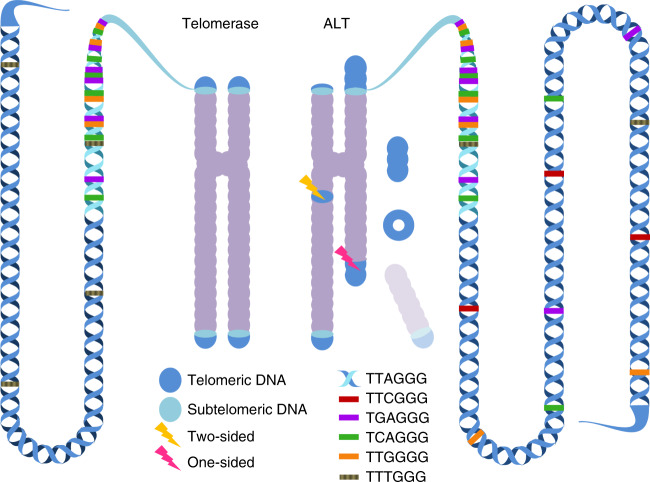
Genomic footprints of telomerase-mediated telomere elongation and ALT. It is known that telomeres elongated by telomerase have a homologous length with few TVRs in distal telomeric regions (left), while ALT telomeres have heterogeneous lengths with an increased amount of TVRs (right). Moreover, ALT cells have abundant extrachromosomal telomeric sequences. From this study, we conclude that the chromosomes of ALT cells have a higher number of aberrant interstitial telomere insertions, most of which are one-sided and accompanied by a loss of the adjacent chromosomal segment. We also showed that several TVRs occurring as singletons are more abundant in ALT telomeres, while one singleton (TTTGGG) was more abundant in telomerase-elongated telomeres. Please note that it is currently undetermined whether the different types of singletons are located in proximal or distal telomeric regions.

In this study, we have shown that the presence of ALT-associated mutations in tumors correlates with increased telomere content, enrichment of isolated TVRs in t-type context (singletons), a higher number of genomic breakpoints, and intrachromosomal telomere insertions (Fig. 5). In contrast, tumors with mutations associated with a possible telomerase activation showed moderate decrease of telomere content and increased *TERT* expression. Hence, *TERT* reactivation may not suffice to fully counteract the telomere loss associated with high proliferation and/or occur when advanced telomere attrition increases the selective pressure to activate telomere maintenance. The observed telomere content increase in ATRX/DAXX^trunc^ versus the decrease in TERT^mod^ samples is in agreement with the recent findings of Barthel et al.^26^. The higher telomere content in ATRX/DAXX^trunc^ tumors indicates that the negative feedback loop that constrains telomere elongation to a physiological level in healthy telomerase-expressing cells^40,41^ is bypassed by the ALT process, while it seems to remain intact in telomerase-positive tumors. In addition to telomere elongation, the increase of telomere content in ALT-positive tumors detected by sequencing-based methods may partly stem from aberrant intrachromosomal telomere insertions^18^ or extrachromosomal telomeric DNA^42^. Although almost all tumors must maintain their telomeres^43^, we only detected somatic mutations highly associated with ALT or telomerase activation in a subset of the samples. In tumors arising from tissues with high rates of self-renewal, telomerase is likely to already be epigenetically activated in the cell of origin^44,45^. Thus, telomere maintenance activating mutations occur more frequently in tumors derived from slowly replicating cells^46^. In line with this assumption, we observed high rates of TMM-associated mutations in brain, liver, bladder, and kidney tumors and *TERT* expression despite lack of TMM-associated mutations in lymphomas, tumors of the gastrointestinal tract, and female reproductive system. The exceptions were pilocytic astrocytoma, pancreatic, and prostate adenocarcinoma, which all originate from slowly replicating tissues, but had almost no TMM-associated alterations. In pancreatic and prostate cancer, TERT activity has been detected^47,48^, suggesting other means of telomerase activation. In pilocytic astrocytoma, neither telomerase expression/activity nor ALT was observed, but preALT characteristics have been reported^49,50^. Therefore, a TMM may only be fully activated upon progression of this slow-growing tumor type. Medulloblastoma samples only had a TERT^mod^ frequency of 14% and one of the lowest average telomere contents in our study. While TERTp^mut^ tend to occur in older patients of the SHH subgroup, the *TERT* promoter is frequently methylated in younger SHH patients and other medulloblastoma subgroups^51^. Interestingly, SHH medulloblastomas are thought to arise from granule neuron precursor cells^52^. This is a cell type with an extremely high rate of turnover during development and infancy, which may explain the *TERT* promoter methylation in younger SHH patients. In agreement with data suggesting that *TERT* expression is higher in TERTp^mut^ than in *TERT* promoter methylated medulloblastomas^51^, we found that the telomere content was significantly higher in medulloblastomas with TERT^mod^ than in those without (*p* = 0.0045, Wilcoxon rank-sum test).

In our study, we systematically mapped telomere insertions into nontelomeric genomic regions using WGS data. They were most frequently accompanied by a loss of the adjacent chromosomal segment or located at copy-number neutral sites. Surprisingly, the latter telomere insertions were rarely two-sided and chromothripsis or other structural variations in the adjacent genomic regions occurred only in about half of the cases. As broken chromosome ends are highly unstable, the remaining segments must have undetected structural rearrangements, such as subclonal copy number changes or undetected DNA fusions. Taken together, the results suggest that we observe telomere healing or capture^20,21^ rather than telomere insertions followed by chromosomal instabilities^18,53^. As microhomology around telomere insertion sites was frequent, the sequences were probably inserted by nonhomologous end-joining^54^ or a microhomology-mediated mechanism^55^.

Telomere insertions were particularly frequent in ATRX/DAXX^trunc^ tumors, in which the abundant extrachromosomal telomeric DNA expands the telomere template pool for microhomology-mediated double-strand repair. We speculate that in this cellular environment, a high load of genomic breakpoints subsequently leads to the observed disproportionately increased number of telomere capture-like events. Due to the stochastic nature of ALT, the likelihood of telomere crisis is elevated, an event that can induce BFB cycles^56,57^ and chromothripsis^58^. Nevertheless, ALT can also stabilize telomeres, which has been shown to counteract genomic instability in certain instances^59^. Either scenario may account for the higher prevalence of chromothripsis and BFB events in ATRX/DAXX^trunc^ cases observed in this study. Together with the correlation of telomere insertions and mutations in *TP53*, *RB1*, *MEN1*, *ATRX*, and *DAXX*, these findings suggest that genome instability and the ALT phenotype are prerequisites for a high number of telomere insertions. Mutations in *TP53*, *RB1*, and *MEN1* have been associated with impaired DNA damage response and repair^60–62^. This may make telomere crisis and genomic rearrangements more likely, while at the same time preventing apoptosis or senescence. Supporting all of these associations, an increased incidence of ALT in combination with chromothripsis was observed in SHH medulloblastomas with *TP53* germline or somatic mutations^59^.

Telomere elongation by ALT or telomerase enriches distinct TVRs^15^. Here, we report a stronger association of singleton TVRs with ATRX/DAXX^trunc^ mutations than TVRs in an arbitrary context. The increase of TVRs has been attributed to the inclusion of subtelomeric regions during ALT via homologous recombination^14^. Whether telomeric sequences with lower TVR density are under positive selection or regions with higher TVR density are under negative selection remains to be clarified.

A possible function for TVRs has been reported in ALT-positive cell lines, where TCAGGG repeats that recruit nuclear receptors were enriched^14,18^. This enrichment was confirmed in a subset of primary ATRX/DAXX^trunc^ tumor samples in our study. However, we found a more pronounced enrichment of TTCGGG or TGAGGG. While TGAGGG has previously been associated with ALT^14^, the high prevalence of TTCGGG singletons in ALT is a novel discovery. No proteins with strong affinity to these two TVRs are currently known. This may indicate a more passive mode of action, for instance deprotection of telomeres by shelterin displacement^14^, and/or alteration of the telomeric G-quadruplex conformation^15^. Notably, we report for the first time that TTTGGG singletons but not TTTGGG in arbitrary context were depleted in ATRX/DAXX^trunc^ samples. This finding underlines the necessity to consider the sequence context of TVRs. None of the current models of ALT provide an explanation for this specific TVR depletion.

The methodologies presented here expand the established telomere content estimation from genomic sequencing by the context-dependent analysis of TVRs and telomere insertions. By applying them within a large-scale pan-cancer study, we provide a valuable resource for the further characterization of different TMMs in cancer cells based on WGS data.

## Methods

### Sequencing data

WGS and expression data were obtained from the ICGC/TCGA PCAWG project^23^. The WGS reads of tumor and control samples were aligned with bwa-mem by the PCAWG-tech group. Control samples were usually blood. In a small number of cases, the controls were obtained from tumor-adjacent or other tissue^23^. Tumors with multiple samples were excluded from this study, as well as one sample pair with reads shorter than 30 bp. Expression data was in the format of normalized RNA read counts per gene and only available for 1033 of 2519 patients. RNA sequencing BAM files aligned with STAR by the PCAWG-tech group were available for 867 tumor samples. To avoid confusion, we used the name “(central nervous system) CNS-LGG” (lower grade glioma, i.e., grades II and III) for the “CNS-Oligo” tumor type, because several samples in this cohort did not have the genetic markers (i.e., 1p/19q co-deletions) for oligodendroglioma required by the WHO^63^. A detailed overview of tumor type abbreviations with the included subtypes is given in Supplementary Table 4.

### Mutation data

Somatic simple nucleotide, structural variations, and copy numbers were obtained from the PCAWG consensus calls (Synapse IDs syn7364923, syn7596712, and syn8042992, respectively). Structural variations were not available for 24 tumor samples.

### Telomere read extraction and computational telomere content estimation

The telomere content of WGS samples was determined using the software tool TelomereHunter^64^. In short, telomeric reads containing six nonconsecutive instances of the four most common telomeric repeat types (TTAGGG, TCAGGG, TGAGGG, and TTGGGG) were extracted. For the further analysis, only unmapped reads or reads with a very low alignment confidence (mapping quality lower than 8) were considered. The telomere content was determined by normalizing the telomere read count by all reads in the sample with a GC content of 48–52%.

### Determining TMM-associated mutations

Samples with a truncating *ATRX* or *DAXX* alteration (frame-shift insertion/deletion, stop-codon gain, or structural variation breakpoint within the gene) were defined as ATRX/DAXX^trunc^, samples with other simple nucleotide variants were defined as ATRX/DAXX^non-trunc^. Deletions that only affected intronic regions of *ATRX* were not considered. Of note, a frame-shift deletion called in the tumor sample of donor SP112201 was excluded as a false positive after visual inspection in the Integrative Genomics Viewer (IGV)^65,66^. Samples with a structural variation breakpoint on the plus strand 20 kb upstream of *TERT* the TSS were defined as TERTp^SV^. TERT^amp^ samples had at least six additional copies of the *TERT* gene compared to the mean ploidy of the sample. Tumor samples with a C228T or C250T *TERT* promoter mutation were defined as TERTp^mut^. Due to the low sequencing coverage at the *TERT* promoter, these mutations were called using less stringent criteria (at least two reads with the mutated base, mutational frequency of at least 20%) in addition to the PCAWG consensus single nucleotide variant (SNV) calls (Synapse ID syn7364923). If multiple of these *TERT* modifications were present, the sample was defined as TERT^mult^. Samples with these *TERT* alterations were summarized as TERT^mod^. Samples without any of these alterations were defined as “wild type”. If a sample had both a TERT^mod^ alteration and an *ATRX/DAXX* alteration, it was defined as “mixed”. For some analyses, ATRX/DAXX^non-trunc^, mixed and wild-type samples were summarized as “other”.

### Overlap of juxtaposed positions upstream of *TERT* and predicted super-enhancers

For the closest structural variation (SV) of each tumor sample to the *TERT* TSS, the juxtaposed genomic coordinates were compared to 65,950 predicted super-enhancers from the dbSUPER database^30^. Only SVs on the plus strand and within 1 mb of the *TERT* TSS were considered. Overlaps of juxtaposed positions with super-enhancer sites were defined as direct overlaps. Super-enhancer sites within 1 mb of the juxtaposed position were defined as indirect overlaps.

### Telomere insertion detection

To find insertions of telomeric sequences into nontelomeric regions in the genome, we searched for tumor-specific discordant paired-end reads, where one end was an extracted telomere read and the other end was nontelomeric and uniquely mapped to a chromosome (mapping quality > 30). In 1 kb regions containing at least three discordant reads in the tumor sample and none in the matching control, exact positions of telomere insertions were defined by at least three split reads spanning the insertion site. The split reads had to contain at least one TTAGGG repeat. Regions with discordant read pairs in at least 15 control samples were excluded. Finally, the insertion sites were visualized using IGV^65,66^ to identify and remove remaining false positives. A telomere insertion was defined as two-sided if another telomere insertion in opposite orientation was found in the downstream 10 kb of the reference genome with the same repeat on the forward strand. Otherwise it was defined as one-sided.

### Breakpoint detection

Breakpoints were obtained from the consensus breakpoint list of structural and copy number variation calls (Synapse ID syn8042992). In short, six copy number detection tools were run on all samples, including the consensus structural variations breakpoints. From the obtained chromosomal segments of the individual callers another set of consensus breakpoints was calculated.

### Chromothripsis detection

To identify chromothripsis events, we extended the set of statistical criteria proposed by Korbel and Campbell^67^. The basic idea is to determine whether there is a statistically significant number of interleaved structural variants in a contiguous genomic region. We did this by constructing a graph whose nodes correspond to SVs and whose edges connect interleaved SVs. The identified clusters of SVs were also tested for the presence of alternating copy number and loss-of-heterozygosity patterns. The resulting chromothripsis calls were validated visually. The full description of the methodology and the detailed patterns of chromothripsis events in the genomes are described in a separate study^24^. Only high-confidence chromothripsis calls were included in this analysis.

### BFB detection

At least two fold-back inversions on the same chromosome arm were defined as BFB events. Fold-back inversions had to fulfill the following requirements adapted from Cheng et al.^68^: (1) the two breakpoints of the inversion are less than 20 kb apart; (2) the inversion does not have a reciprocal partner, such that inversion1_start < inversion2_start < inversion1_end < inversion2_end; and (3) there is a copy number change at the inversion site.

### Copy number changes at telomere insertion sites

Copy numbers of chromosomal segments were obtained from the PCAWG consensus calls (Synapse ID syn8042992). Copy numbers reveal gains or losses of chromosomal segments based on coverage and B-allele frequency, but were here limited to segments of at least 10 kb. The breakpoint estimations could differ from the actual site by up to 50 kb. Therefore, telomere insertions were assigned to the closest breakpoint within 50 kb. If there was no breakpoint within 50 kb or the copy numbers at either side of the telomere insertion were the same, the copy number change at the telomere insertion was defined as neutral.

### Structural variations near telomere insertion sites

Structural variation annotation was obtained from the PCAWG consensus calls (Synapse ID syn7596712), which was based on discordant mate pairs and split reads, providing exact breakpoints. Because copy number variations smaller than 10 kb were not detected by copy number callers, small deletions next to the telomere insertion site may be missed. We therefore searched for structural variations within 10 kb of a telomere insertion to detect these cases.

### Candidate gene selection for correlation analysis

A list of 1725 telomere maintenance associated human genes was obtained from TelNet^33^ on February 20, 2017. After removing genes without a unique Ensembl IDs in the GENCODE^69^ v19 HAVANA annotation, the remaining 1686 genes were used for correlation of telomere insertions and simple nucleotide variants.

### TERRA detection

TelomereHunter was run on RNA-Seq BAM files to count reads containing at least *k* nonconsecutive instances of the four most common telomeric repeat types (TTAGGG, TCAGGG, TGAGGG, and TTGGGG). The repeat threshold *k* was chosen depending on the read length: *k* = 7 for 45–50 bp, *k* = 10 for 75–76 bp, and *k* = 14 for 99–101 bp. The resulting TERRA read counts were normalized by the total number of reads in the sample. For better readability, this number was multiplied by 1 Mio.

### Detection of TVRs

TVRs were detected by searching for hexamers of the type NNNGGG in the extracted telomere reads. Each base was required to have a base quality of at least 20. The neighboring 18 bp on either side of the TVR were determined. For further analysis, NNNGGG TVRs were once computed for arbitrary context and once for t-type context ((TTAGGG)_3_-NNNGGG-(TTAGGG)_3_, also called “singletons”). The absolute counts were normalized to the total number of reads in the sample. The expected pattern counts in arbitrary context were calculated as: telomere content tumor/control log2 ratio. The expected singleton counts at different telomere content tumor/control log2 ratios were taken from the regression line through TERT^mod^ samples. The singleton occurrence heatmap was generated using the R package ComplexHeatmap^70^.

### Classifier for predicting active TMMs

A random forest classifier to distinguish ATRX/DAXX^trunc^ and TERT^mod^ samples was built using the R packages “randomForest”^71^ and “caret”^72^ with the following eight features: telomere content tumor/control log2 ratio, number of telomere insertions, number of breakpoints, and the distance of TGAGGG, TCAGGG, TTGGGG, TTCGGG, and TTTGGG singletons (i.e., repeats in a t-type context) to their expected occurrence. To deal with the imbalance in the data set (i.e., 266 TERT^mod^ samples versus 63 ATRX/DAXX^trunc^ samples without missing data), the model was trained with a down-sampled training set. The performance was determined using 10-fold cross-validation.

### Statistics

Differences between ATRX/DAXX^trunc^ and TERT^mod^ samples in terms of telomere content, percent breakpoints with telomere insertions, and singleton repeat abundance were tested using two-sided Wilcoxon rank-sum tests. Singleton repeat abundance *p*-values were corrected for multiple testing using the Bonferroni method. To reduce the influence of outliers, correlation coefficients were calculated with the Spearman method. Correlation between control telomere content and age as well as tumor and control telomere content was tested with linear regression. All statistical analyses were carried out using R (R Foundation for Statistical Computing).

### Reporting summary

Further information on research design is available in the Nature Research Reporting Summary linked to this article.

## Supplementary information

Supplementary Information

Description of Additional Supplementary Files

Supplementary Data 1

Supplementary Data 2

Reporting Summary

## Data Availability

Somatic and germline variant calls, mutational signatures, subclonal reconstructions, transcript abundance, splice calls, and other core data generated by the ICGC/TCGA PCAWG Consortium is described here^23^ and available for download at [https://dcc.icgc.org/releases/PCAWG]. Additional information on accessing the data, including raw read files, can be found at [https://docs.icgc.org/pcawg/data/]. In accordance with the data access policies of the ICGC and TCGA projects, most molecular, clinical and specimen data are in an open tier which does not require access approval. To access potentially identification information, such as germline alleles and underlying sequencing data, researchers will need to apply to the TCGA Data Access Committee (DAC) via dbGaP for access to the TCGA portion of the dataset, and to the ICGC Data Access Compliance Office (DACO) for the ICGC portion. In addition, to access somatic SNVs derived from TCGA donors, researchers will also need to obtain dbGaP authorization. Derived data sets described specifically used in this manuscript are catalogued on Synapse. Data access is possible via the ICGC data portal (DCC), were the original files are split into ICGC and TCGA subsets due to different access regulations: somatic simple nucleotide calls (syn7364923): and [https://dcc.icgc.org/releases/PCAWG/consensus_snv_indel], structural variation calls (syn7596712): and [https://dcc.icgc.org/releases/PCAWG/consensus_sv], copy number calls (syn8042992): and [https://dcc.icgc.org/releases/PCAWG/consensus_cnv].

## Code availability

The software TelomereHunter used for the in silico analysis of the genomic footprints of activated TMMs is available from [https://www.dkfz.de/en/applied-bioinformatics/telomerehunter/telomerehunter.html]. The core computational pipelines used by the PCAWG Consortium for alignment, quality control, and variant calling are available to the public at https://dockstore.org/search?search=pcawg under the GNU General Public License v3.0, which allows for reuse and distribution.
