# Supplementary material for: Genomic footprints of activated telomere maintenance mechanisms in cancer
Source: Nat Commun. 2020 Feb 5;11:733. doi: 10.1038/s41467-019-13824-9 (PMC7002710; doi:10.1038/s41467-019-13824-9)
Supplement: Supplementary file 2 — Description of Additional Supplementary Files [file 41467_2019_13824_MOESM2_ESM.pdf]

Description of Additional Supplementary Files

File name: Supplementary Data 1
Description: Sample information. Information on tumor type, data availability and telomere-associated information of all samples is given. "Singleton_dist" column describe the divergence of observed singleton repeat counts to expected counts. The ALT probability was calculated by the random forest classifier described in the study.

File name: Supplementary Data 2
Description: Most frequent neighboring sequences of telomere repeat variants. For each pattern, the top three telomere variant repeat sequence contexts (18 bp on either side) and their percentage in all samples, ATRX/DAXX^{trunc} samples and TERT^{mod} samples are shown.
